# Dexamethasone disrupts intracellular pH homeostasis to delay coronavirus infectious bronchitis virus cell entry via sodium hydrogen exchanger 3 activation

**DOI:** 10.1128/jvi.01894-24

**Published:** 2025-05-09

**Authors:** Jun Dai, Yiyi Feng, Hong Long, Ying Liao, Lei Tan, Yingjie Sun, Cuiping Song, Xusheng Qiu, Chan Ding

**Affiliations:** 1 Shanghai Veterinary Research Institute, Chinese Academy of Agricultural Sciences 118161, Shanghai, China; 2 Experimental Animal Center, Zunyi Medical University 66367, Zunyi, China; 3 Jiangsu Co-innovation Center for Prevention and Control of Important Animal Infectious Diseases and Zoonoses, Yangzhou University 38043, Yangzhou, China; University of Kentucky College of Medicine, Lexington, Kentucky, USA

**Keywords:** dexamethasone, coronavirus, infectious bronchitis virus, Na^+^/H^+^exchanger 3, pH

## Abstract

Coronavirus entry into host cells enables the virus to initiate its replication cycle efficiently while evading host immune response. Cell entry is intricately associated with pH levels in the cytoplasm or endosomes. In this study, we observed that the sodium hydrogen exchanger 3 (Na^+^/H^+^ exchanger 3 or NHE3), which is strongly activated by dexamethasone (Dex) to promote cell membrane Na^+^/H^+^ exchange, was critical for cytoplasmic and endosomal acidification. Dex activates NHE3, which increases intracellular pH and blocks the initiation of coronavirus infectious bronchitis virus (IBV) negative-stranded genomic RNA synthesis. Also, Dex antiviral effects are relieved by the glucocorticoid receptor (GR) antagonist RU486 and the NHE3 selective inhibitor tenapanor. These results show that Dex antiviral effects depend on GR and NHE3 activities. Furthermore, Dex exhibits remarkable dose-dependent inhibition of IBV replication, although its antiviral effects are constrained by specific virus and cell types. To our knowledge, this is the first report to show that Dex helps suppress the entry of coronavirus IBV into cells by promoting proton leak pathways, as well as by precisely tuning luminal pH levels mediated by NHE3. Disrupted cytoplasmic pH homeostasis, triggered by Dex and NHE3, plays a crucial role in impeding coronavirus IBV replication. Therefore, cytoplasmic pH plays an essential role during IBV cell entry, probably assisting viruses at the fusion and/or uncoating stages. The strategic modulation of NHE3 activity to regulate intracellular pH could provide a compelling mechanism when developing potent anti-coronavirus drugs.

**IMPORTANCE:**

Since the outbreak of coronavirus disease 2019, dexamethasone (Dex) has been proven to be the first drug that can reduce the mortality rate of coronavirus patients to a certain extent, but its antiviral effect is limited and its underlying mechanism has not been fully clarified. Here, we comprehensively evaluated the effect of Dex on coronavirus infectious bronchitis virus (IBV) replication and found that the antiviral effect of Dex is achieved by regulating sodium hydrogen exchanger 3 (NHE3) activity through the influence of glucocorticoid receptor on cytoplasmic pH or endosome pH. Dex activates NHE3, leading to an increase in intracellular pH and blocking the initiation of negative-stranded genomic RNA synthesis of coronavirus IBV. In this study, we identified the mechanism by which glucocorticoids counteract coronaviruses in cell models, laying the foundation for the development of novel antiviral drugs.

## INTRODUCTION

Coronavirus cell entry is key for virus replication cycles and evading host antiviral responses (1, 2), which was previously reviewed by our group (3). Briefly, coronavirus enters host cells mainly via plasma membrane fusion or endocytosis (4, 5). Its spike (S) glycoprotein mediates viral entry through pH-dependent endocytosis (6–8), which may occur at either the plasma membrane (early pathway) or the endosomal membrane (late pathway), depending on the cell type (5, 9, 10). The cell entry of most coronaviruses—e.g., infectious bronchitis virus (IBV) (11), porcine deltacoronavirus (PDCoV) (12, 13), porcine hemagglutinating encephalomyelitis virus (14), porcine epidemic diarrhea virus (PEDV) (15), murine coronavirus (16, 17), transmissible gastroenteritis virus (TGEV) (18), and feline coronaviruses (19)—occurs in a low-pH-independent manner and is sensitive to endosomes/lysosome pH (8). As acidic pH in endosomal compartments triggers conformational changes in the S protein to mediate fusion (17, 20, 21), this process facilitates viral genomic RNA release into the cytoplasm to initiate viral replication (2, 5). Similarly, a high lysosomal pH impairs virus degradation in lysosomes (22, 23). Links between organellar pH and coronavirus entry have been postulated (24). A pH-dependent cell entry mechanism was reported for severe acute respiratory syndrome coronavirus (SARS-CoV) after DC-SIGN (dendritic cell-specific intercellular adhesion molecule-3-grabbing nonintegrin) receptor binding (6). Importantly, coronaviruses exploit host endosomal acidification to accommodate viral replication requirements, and they exploit low-pH endosomal environments for immune evasion (21, 23, 25, 26).

Previous studies have reported crucial roles for sodium hydrogen (NA^+^/H^+^) exchangers (NHEs) in maintaining proton balance in endosomes, which are precisely coordinated with V-ATPase to facilitate endosome acidification and maturation (27–31). NHEs help maintain the intracellular pH within physiological limits, and the intracellular pH is increased by NHE activation (32). The molecules, mechanisms, and physiology of the individual NHE isoforms can be found in the previous review (33). NHE3 is encoded by *SLC9A3* and is representative of the NHE isoforms which continually traffic between the recycling system and the plasma membrane, with their major functions occurring at the plasma membrane (34–38). The recycling regulation of NHE3 in cells can be found in the previous review (35). Moreover, NHE3 has a significant influence over cell receptor-mediated endocytosis, actively participating in endosomal acidification and maintaining cytoplasmic pH homeostasis (28, 39–41). Similarly, NHE3 inhibition disturbs endosomal acidification and slows down endocytosis (28).

Endosomal Na^+^/H^+^ exchangers may regulate key host antiviral defense mechanisms and mediators that act to drive inflammatory organ injury (42). Currently, causal links between endosomal luminal pH and coronavirus entry remain unclear. The prevailing endosomal pH model overwhelmingly focuses on the proton pumping as mediated by V-ATPase, while proton leak pathways for precise luminal pH tuning and coronavirus entry, putatively mediated by NHE3, remain largely understudied (27, 43). In this study, we showed that NHE3 activation was closely related to coronavirus pathogenicity. For example, TGEV and PEDV infections in piglets suppress NHE3 activity on intestinal surfaces, thereby impacting Na^+^/H^+^ exchange and electrolyte absorption in intestinal cells (44–48), which contributes to piglet diarrhea. Additionally, a genome-wide small interfering RNA screen showed that NHE3 was required for efficient lymphocytic choriomeningitis virus multiplication in HeLa cells (49). Notably, SARS-CoV-2 interactions with NHE3 in intestinal tissues differ from those in other tissues. Following viral infection, a cytokine storm may be generated by disrupting the gut microbiota and inhibiting NHE3 (32).

Presently, there is a dearth of evidence on the impact of NHE3 activity on viral replication. Previous studies have reported that acute NHE3 activation by dexamethasone (Dex) is closely associated with serum- and glucocorticoid-inducible kinase 1 (SGK1) activation and subsequent NHE3 phosphorylation at Ser663 (50), processes requiring a functional glucocorticoid receptor (GR) (51). To our knowledge, no studies have explored correlations between IBV replication and NHE3 activity. Interestingly, we have confirmed that Dex activates NHE3, which increases intracellular pH and blocks coronavirus IBV genome replication initiation, while Dex antiviral effects are relieved by GR antagonist RU486 and the NHE3 selective inhibitor tenapanor. Thus, Dex activation may enhance NHE3 activity, which in turn upregulates intracellular pH and hinders IBV genome replication initiation. Cytoplasmic and endosomal pH have important roles in coronavirus entry, which is significantly associated with human coronavirus spread. Therefore, targeting NHE3 activity to regulate intracellular pH could be promising for antiviral drug development against coronaviruses. Additionally, Dex is the first drug to show life-saving efficacy in patients with coronavirus disease 2019 (COVID-19) (52). However, controversy prevails regarding glucocorticoid use in treating coronavirus. While Dex may modulate inflammation-mediated lung injury and thereby reduce progression to respiratory failure and death (53–56), the glucocorticoid also expands immunosuppressive neutrophils and repatterns their interactions in COVID-19 (57). Thus, glucocorticoid use may be a double-edged sword. In this study, we identified a mechanism whereby glucocorticoids counteracted coronaviruses in cellular models, laying the foundation for novel drug development against coronaviruses.

## RESULTS

### Dex suppresses IBV replication in a dose-dependent manner

**Fig 1:**
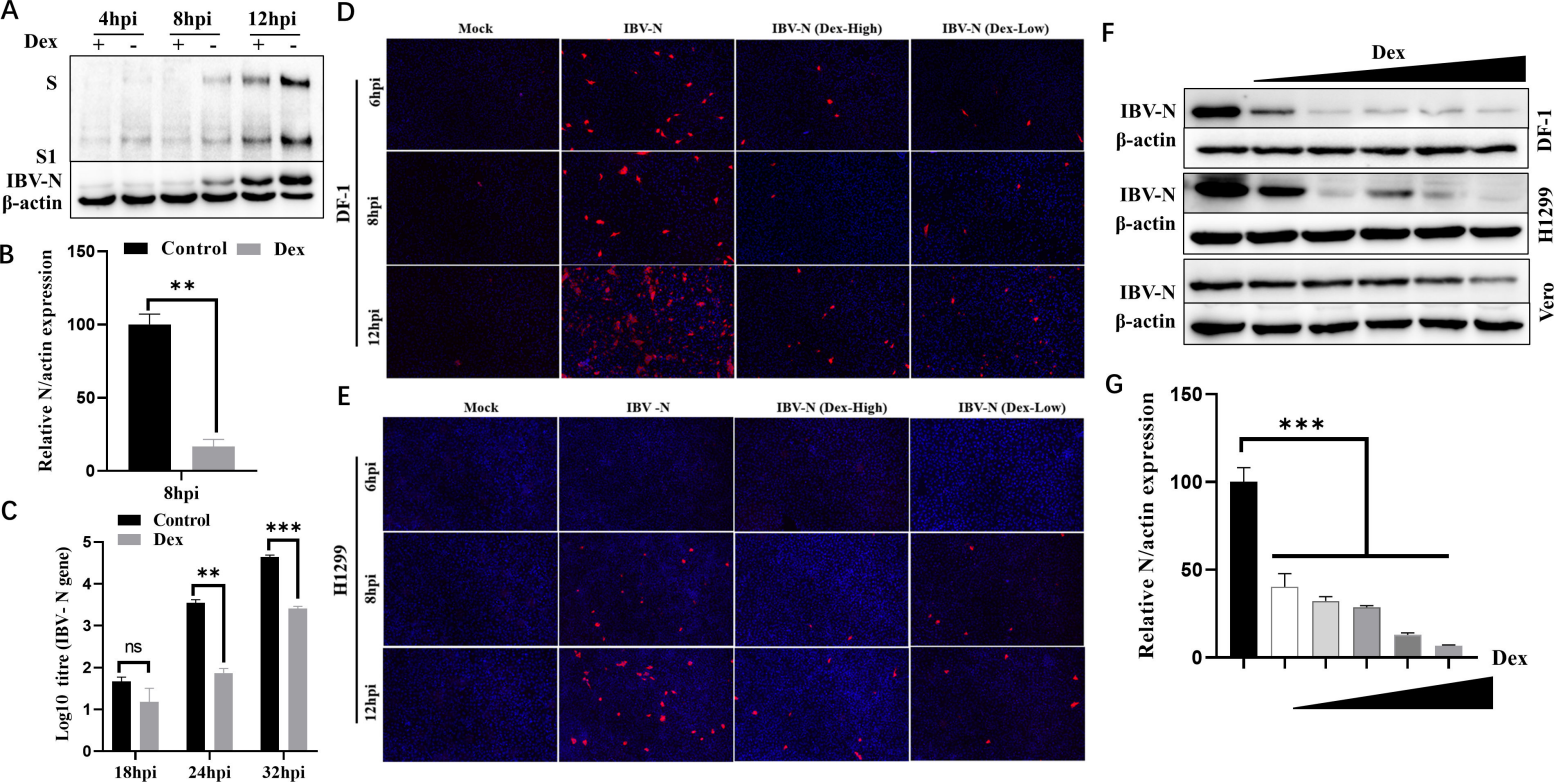
IBV replication is strongly inhibited in cells by pretreatment with Dex. (**A**) DF-1 cells were pretreated with Dex (10μg/mL) for 12 h and then infected with IBV at a multiplicity of infection (MOI) of 1. Cells were collected at 4 hpi, 8 hpi, and 12 hpi to determine IBV-N, IBV-S, and IBV-S1 protein levels by western blotting. (**B**) DF-1 cells were pretreated with Dex (10 µg/mL) for 12 h and then infected with IBV (MOI = 1). Cells were harvested at 8 hpi, and *IBV-N* mRNA levels were determined by qRT-PCR. (**C**) DF-1 cells were pretreated with Dex (10 µg/mL) for 12 h and then infected with IBV (MOI = 1). Cell supernatants were harvested at 18 hpi, 24 hpi, and 32 hpi to determine *IBV-N* mRNA levels using absolute qRT-PCR. DF-1 (D) and H1299 (**E**) cells were pretreated with high (100 µg/mL) or low (0.01 µg/mL) Dex concentrations for 12 h. Cells were then infected with IBV (MOI = 1). Immunofluorescence was performed at 6, 8, and 12 hpi using anti-IBV-N antibodies. (**F**) DF-1, H1299, and Vero cells were pretreated with different Dex concentrations (0 μg/mL–100 μg/mL) for 12 h and then infected with IBV (MOI = 1). Cells were collected at 8 hpi to determine IBV-N protein levels by western blotting. (**G**) DF-1 cells were pretreated with different Dex concentrations (0 μg/mL–100 μg/mL) for 12 h and then infected with IBV (MOI = 1). Cells were harvested at 8 hpi to determine *IBV-N* mRNA levels by qRT-PCR.

Recent research reported that Dex possessed a remarkable binding affinity toward multiple sites on the SARS-CoV-2 spike 1 protein (S1) (58, 59). This binding activity cooperatively inhibited interactions between S1 and angiotensin-converting enzyme 2, thereby effectively blocking virus entry (58, 59). To investigate whether Dex affected coronavirus IBV replication in an S1-binding-independent manner, DF-1 and H1299 cells were infected with IBV and subjected to Dex treatment prior to virus infection. IBV infection in DF-1 cells was significantly inhibited by Dex when compared to the Dex-untreated cells (Fig. 1A). To confirm that Dex inhibited IBV replication, we performed quantitative PCR (qPCR) and immunofluorescence assay to detect viral mRNA and protein levels, respectively. As expected, viral mRNA levels in intracellular and cell supernatants were significantly inhibited by Dex (Fig. 1B and C), with similar results for virus protein levels (Fig. 1D and E). Furthermore, we identified a dose-dependent inhibition of IBV replication upon Dex treatment (Fig. 1F and G). Therefore, Dex significantly inhibited coronavirus IBV replication and exhibited a significant dose-dependent effect.

### Dex antiviral activity is related to virus and cell type

**Fig 2:**
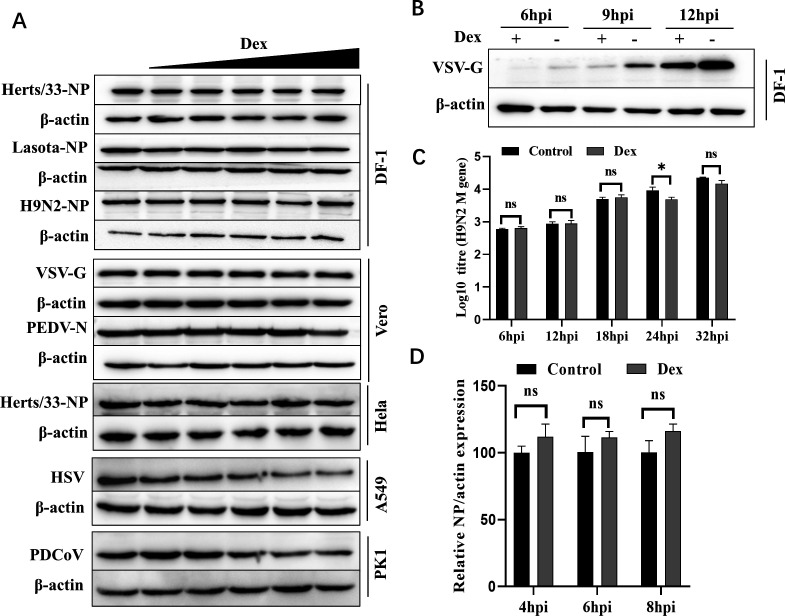
Dex’s antiviral effects are constrained by specific virus and cell types. (**A**) DF-1, Vero, HeLa, A549, and PK1 cells were pretreated with different Dex concentrations (0 μg/mL–100 μg/mL) for 12 h, and then infected (MOI = 1) with Herts/33, LaSota, H9N2, VSV, PEDV, HSV, and PDCoV. Cells were collected at 8 hpi for western blotting. Dex-untreated cells were used as controls. (**B**) DF-1 cells were pretreated with Dex (100 µg/mL) for 12 h and then infected with VSV (MOI = 1). Cells were collected at 6, 9, and 12 hpi for western blotting. Dex-untreated cells were used as controls. (**C**) DF-1 cells were pretreated with Dex (10 µg/mL) for 12 h and then infected with H9N2 (MOI = 0.01). Cells were harvested at indicated times for absolute qPCR analysis of viral RNA levels (*M* gene). (**D**) DF-1 cells were pretreated with Dex (10 µg/mL) for 12 h, after which cells were infected with Herts/33 (MOI = 1). Cells were harvested at indicated times for qPCR analysis of *NDV-NP* RNA levels.

We observed a discrepancy in Dex antiviral effects toward IBV in DF-1, H1299, and Vero cells (Fig. 1F and G). Therefore, we investigated if this activity was related to virus and cell type. To this end, the impact of Dex on multiple virus replications was examined and showed that Dex antiviral activity varied depending on virus and cell type. For instance, Dex significantly inhibited IBV, herpes simplex virus (HSV), vesicular stomatitis virus (VSV), and PDCoV replication, whereas its antiviral effects toward Newcastle disease virus (NDV) (Herts/33 and LaSota), H9N2, and PEDV were non-significant (Fig. 1 and 2). Additionally, the impact of Dex on IBV and VSV replication was also related to cell type. For example, when treating DF-1, H1299, and Vero cells with the same concentration of Dex, the antiviral effect of Dex on IBV was most significant in DF-1 cells, followed by H1299 cells, and the weakest in Vero cells (Fig. 1). Similarly, the antiviral effect of Dex on VSV is most significant on DF-1 cells (Fig. 2B), but not on Vero cells (Fig. 2A). High concentrations of Dex have a certain antiviral effect on HSV and PDCoV, but have almost no antiviral effect on the highly virulent NDV Herts/33 strain (Fig. 2A and D), the nonvirulent NDV LaSota strain (Fig. 2A), and H9N2 (Fig. 2A and C). Thus, Dex antiviral properties exhibited a distinct selectivity and were closely related to specific virus strain and host cell.

### Dex blocks the synthesis initiation of IBV negative-strand genomic RNA, which affects subsequent viral translation

**Fig 3:**
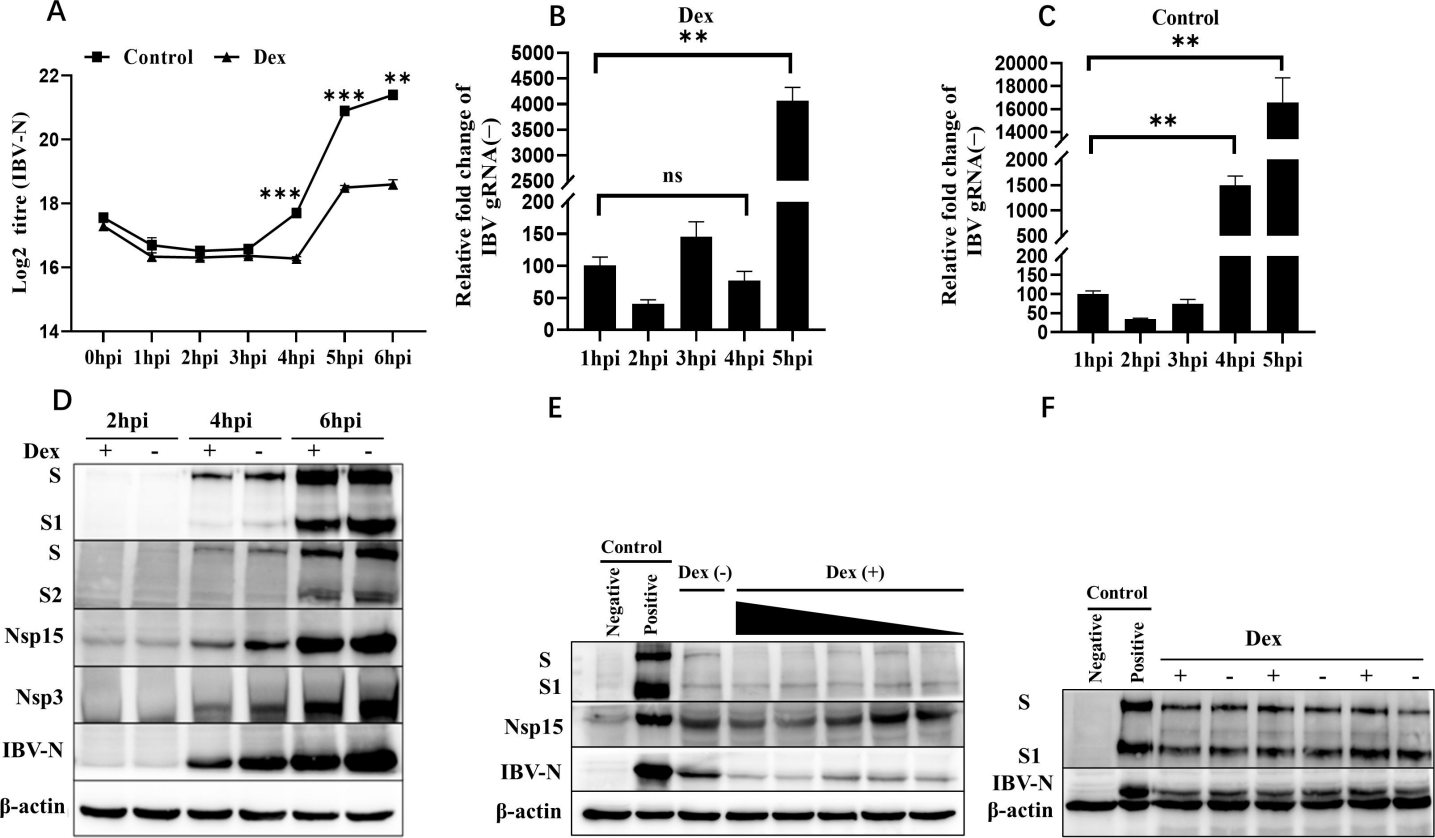
Dex delays IBV cell entry by blocking the initiation of viral negative-strand genomic RNA. (**A**) DF-1 cells were pretreated with Dex (100 µg/mL) for 12 h and then infected with IBV (MOI = 10), incubated at 4°C for 1 h, then transferred to 37°C. Cells were harvested at indicated times for absolute qPCR to determine *IBV-N* mRNA levels. (**B**) DF-1 cells were pretreated with Dex (100 µg/mL) for 12 h and then infected with IBV (MOI = 10). Cells were harvested at indicated times for relative qPCR to determine IBV negative-strand genomic RNA levels. Dex-untreated cells were used as controls (**C**). (**D**) DF-1 cells in 10 cm cell dishes were pretreated with Dex (100 µg/mL) for 12 h and infected with IBV (MOI = 10). Cells were collected at 2 hpi, 4 hpi, and 6 hpi to determine IBV-S, IBV-S1, IBV-S2, IBV-Nsp15, IBV-Nsp3, and IBV-N protein levels by western blotting. (**E**) DF-1 cells were pretreated with different Dex concentrations (0 μg/mL–100 μg/mL) for 12 h, and then infected with IBV (MOI = 10). Cells were collected at 6 hpi to examine IBV-S, IBV-S1, IBV-Nsp15, and IBV-N protein levels by western blotting. Dex-untreated cells were used as controls. (**F**) DF-1 cells in 10 cm cell dishes were pretreated with Dex (100 µg/mL) for 12 h and then infected with IBV (MOI = 10). Cells were collected at 2.5 hpi to determine IBV-N protein levels by western blotting. Dex-untreated cells were used as controls.

Dex was previously shown to effectively block β-coronavirus SARS-CoV-2 cell entry (58, 59). IBV is the prototype virus of the group 3 coronaviruses in the *Coronaviridae* family (60, 61). To investigate whether IBV cell entry was modulated by Dex, we evaluated *IBV-N* mRNA levels and negative-strand genomic RNA synthesis, and also structural and non-structural protein levels during early IBV replication cycles (0–6 hpi) using qPCR and western blotting. We first discovered that *IBV-N* mRNA levels were significantly inhibited by Dex after 3 hpi in DF-1 cells (Fig. 3A), and Dex treatment did not affect IBV adsorption (0 hpi) in DF-1 and H1299 cells (Fig. S1). When compared to the control group, Dex blocked IBV negative-strand genomic RNA production at 4 hpi (Fig. 3B and C). Additionally, we observed a dose-dependent inhibition of IBV structural (N, S, S1, and S2) and non-structural protein (Nsp3 and Nsp15) translation by Dex (Fig. 3D and E). Importantly, Dex had no effects on IBV structural protein (S, S1, and N) levels at 2.5 hpi, when virus protein translation had not yet been initiated (Fig. 3F). Therefore, Dex inhibited initial IBV replication stages without any impact on virus adsorption and endocytosis. Its main effects appeared to be exerted during the replication phase following IBV endocytosis and prior to translation.

### Dex affects the acidic environment in endo-lysosomes, which blocks cargo delivery to lysosomes

**Fig 4:**
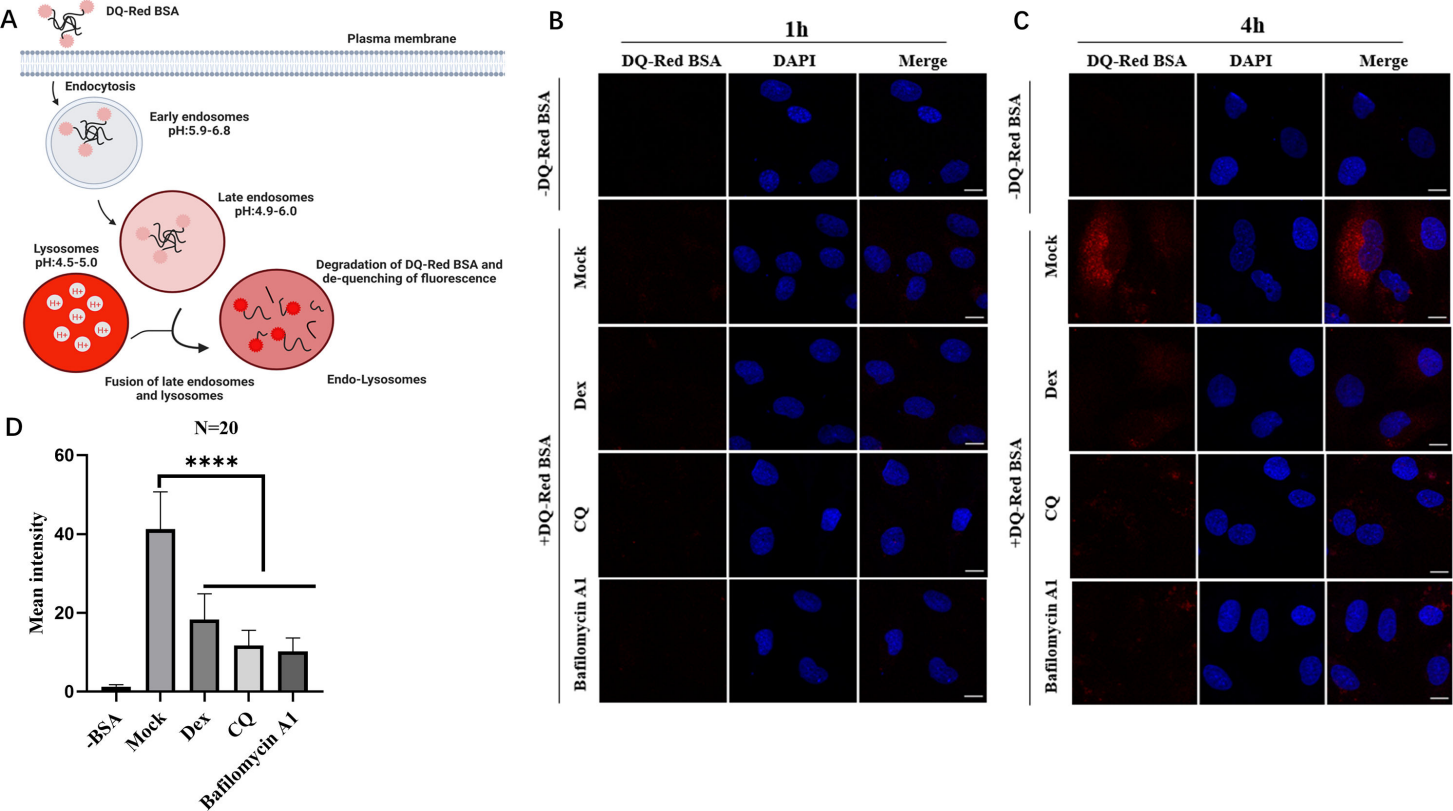
Using DQ-Red BSA trafficking assays to assess the impact of Dex on acidic environments in endo-lysosomes. (**A**) Schematic showing DQ-Red BSA cargo uptake and processing in cells. Representative single-plane confocal micrographs of H1299 cells showing DQ-Red BSA fluorescence (red) after 1 h (**B**) and 4 h (**C**) of uptake. (**D**) Average fluorescence intensity was measured using ImageJ software (*N* = 20). Scale bar = 10 µm.

A low-pH environment in late endosomes or lysosomes may provide important signals for membrane fusion and IBV viral genomic RNA (gRNA) release (62). As described, Dex blocked *IBV-N* mRNA levels and negative-strand genomic RNA synthesis in early infection stages (Fig. 3A through C). Thus, we hypothesized that Dex may have affected the acidic environment in the endo-lysosome system, which blocked IBV membrane fusion and uncoating. To test this, we used the DQ-Red BSA trafficking assays in H1299 cells to assess acidic environment alterations in the endo-lysosomal system due to Dex treatment (63). Mechanistically, DQ-Red BSA degradation in acidic, hydrolase active endo-lysosomes generates small protein fragments that isolate as fluorophores. Hence, de-quenching may be visualized as bright fluorescence in cells (Fig. 4A). As shown (Fig. 4), in mock H1299 cells, bright fluorescence DQ-Red BSA speckles were visible post 4 h uptake when compared to 1 h, indicating delivery to acidic cell compartments (Fig. 4B and C). Compared with the mock group, H1299 cells treated with Dex, chloroquine (CQ), and bafilomycin A generated very weak DQ-Red BSA red fluorescence after 4 h (Fig. 4C and D). Therefore, Dex, similar to CQ and bafilomycin A, putatively interfered with and disrupted acidic environments in the endo-lysosome system, thereby impairing DQ-Red BSA protein hydrolysis and generating weak or absent red fluorescence in cells. Meanwhile, we performed fluorescence imaging using the pH-sensitive probe Protonex Red 600 for quantitative analysis of endosomal and lysosomal pH. As demonstrated in Fig. S2, Dex-treated DF-1 cells exhibited a marked attenuation of red fluorescence intensity compared to mock. This significant decrease in fluorescence signal correlates with alkalinization of acidic compartments, indicating that Dex treatment effectively elevates the luminal pH of both endosomes and lysosomes in DF-1 cells. Hence, the inhibitory impact of Dex toward IBV replication may have arisen from disrupted acidic environments in the endo-lysosome system, blocking IBV delivery to lysosomes.

### Dex restricts IBV in late endosomes

**Fig 5:**
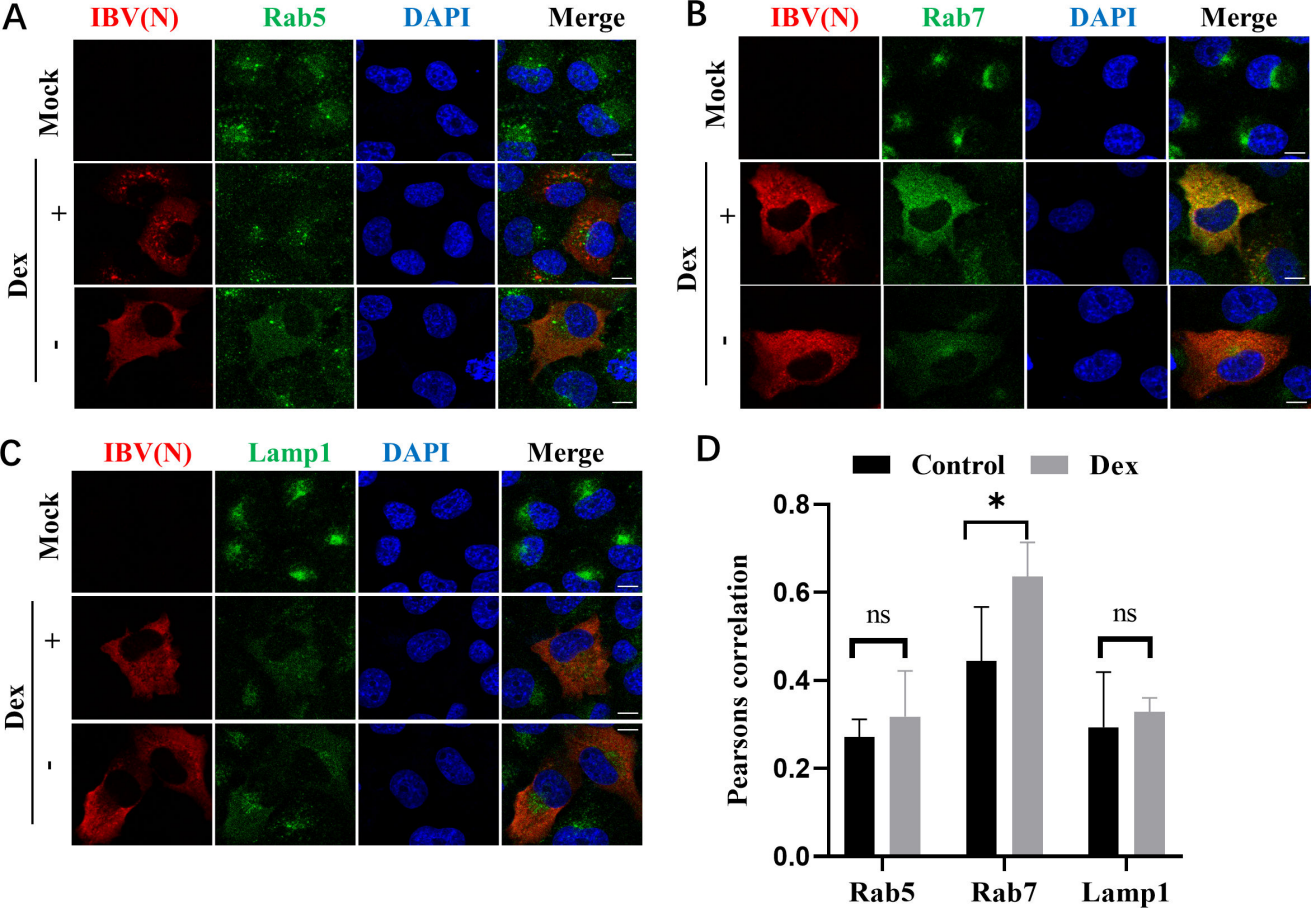
Dex enhances IBV colocalization with late endosomes. H1299 cells were pretreated with Dex (10 µg/mL) for 12 h and then infected with IBV (MOI = 10). Dex-untreated cells were used as controls. Cells were subjected to immunofluorescence with anti-IBV-N (red), anti-Rab5 (green) (**A**), anti-Rab7 (green) (**B**), or anti-LAMP1 (green) (**C**) antibodies at 6 hpi. (**D**) Pearson’s correlation coefficient evaluation of Dex effects on IBV-N protein colocalization with Rab5, Rab7, and Lamp1 in H1299 cells.

We previously showed that IBV tracked along the classical endosome/lysosome pathway and finally fused with late endosome/lysosome (62). In the current study, Dex affected acidic environments in endo-lysosomes, which blocked cargo delivery to lysosomes (Fig. 4). We hypothesized that Dex may have affected these acidic environments in the endo-lysosome system, which blocked IBV membrane fusion or uncoating. To test this, we performed immunofluorescence using specific compartmental markers to examine whether IBV colocalization with early endosomes (Rab5), late endosomes (Rab7), and lysosomes (Lamp1) was influenced by Dex. Interestingly, Dex significantly enhanced IBV-N protein colocalization with late endosomes (Rab7) (Fig. 5B), whereas colocalization with early endosomes (Rab5) and lysosomes (Lamp1) remained mostly unaffected (Fig. 5A and C). Furthermore, Pearson’s correlation analyses revealed a clear colocalization between the IBV-N protein and Rab7 (Fig. 5D). Therefore, Dex potentially restricted IBV to late endosomes.

### Dex-mediated antiviral effects depend on GR and NHE3 activity

**Fig 6:**
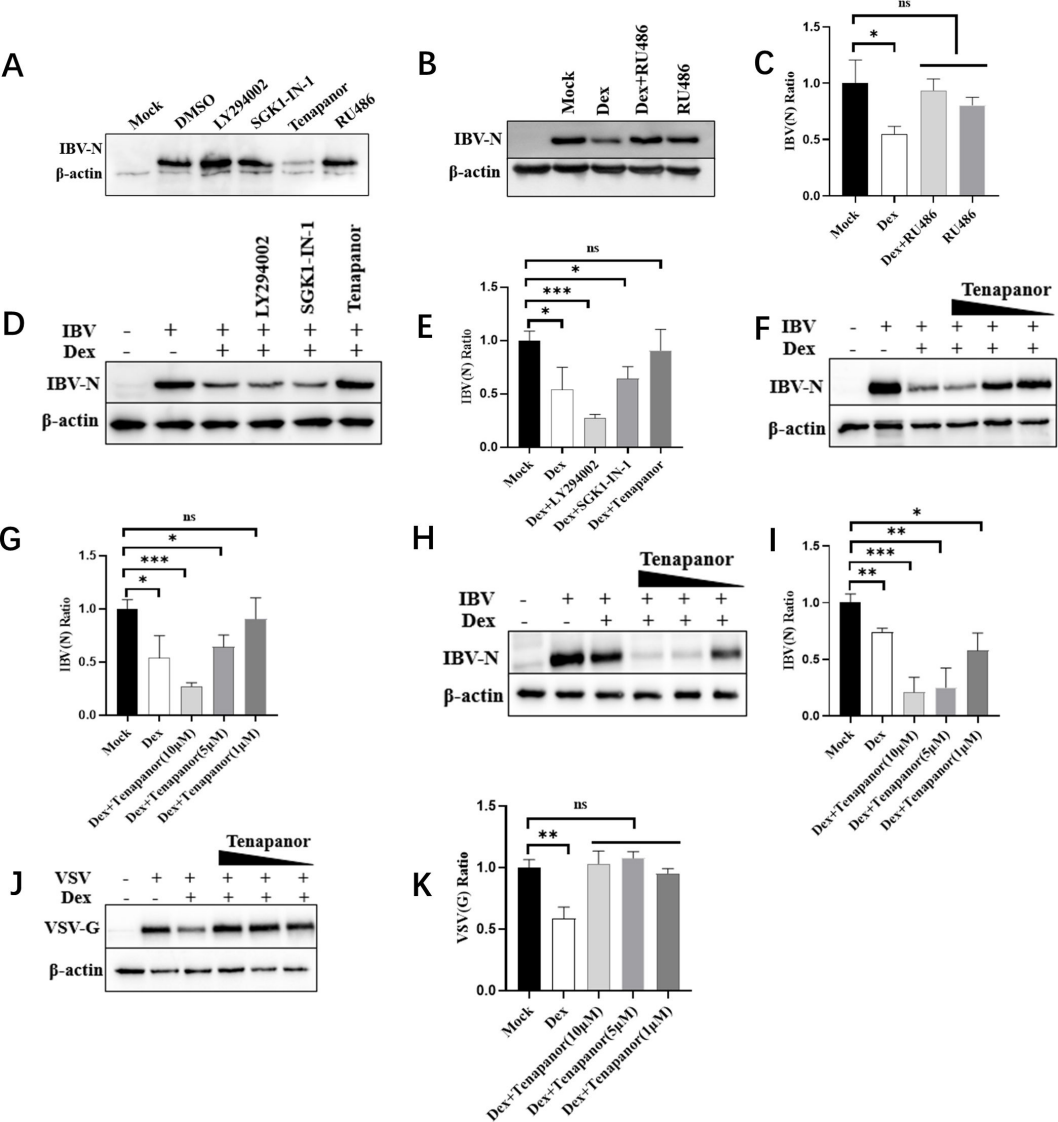
Dex-mediated antiviral effects are abolished by RU486 and tenapanor. (**A**) DF-1 cells were pretreated with dimethyl sulfoxide (DMSO), LY294002 (10 µM), SGK1-IN-1 (10 µM), tenapanor (10 µM), and RU486 (10 µM) for 2 h. After this, cells were infected with IBV (MOI = 1). Cells were collected at 8 hpi, and IBV-N protein levels were determined by western blotting. (**B**) DF-1 cells were pretreated with Dex (10 µg/mL), Dex (10 µg/mL) + RU486 (10 µM), and RU486 (10 µM) for 12 h. After this, cells were infected with IBV (MOI = 1). Cells were collected at 8 hpi, and IBV-N protein levels were determined by western blotting. (**C**) ImageJ software was used for the grayscale scanning of protein bands from western blots (*N* = 3). (**D**) DF-1 cells were pretreated with LY294002 (10 µM), SGK1-IN-1 (10 µM), and tenapanor (10 µM) for 2 h, and then Dex (10 µg/mL) was used to treat cells for 12 h. Cells were collected at 8 hpi, and IBV-N protein levels were determined by western blotting. (**E**) ImageJ software was used for the grayscale scanning of protein bands from western blots (*N* = 3). DF-1 (**F**) and H1299 (**H**) cells were incubated with increasing tenapanor (0 μM–10 μM) concentrations for 2 h, after which cells were treated with Dex (10 µg/mL) for 12 h. Finally, cells were infected with IBV (MOI = 1). Cells were collected at 8 hpi to determine IBV-N protein levels by western blotting. ImageJ software was used for the grayscale scanning of IBV-N protein bands from DF-1 (**G**) and H1299 (**I**) cells. (**J**) DF-1 cells were incubated with increasing tenapanor (0 μM–10 μM) concentrations for 2 h, after which cells were treated with Dex (10 µg/mL) for 12 h. Finally, cells were infected with VSV (MOI = 1). Cells were collected at 8 hpi to determine VSV-G protein levels by western blotting. ImageJ software was used for the grayscale scanning of VSV-G protein bands in DF-1 cells (**K**).

Previous studies reported that Dex interacted with the GR leading to acute NHE3 activation, which was closely related to SGK1 and PI3K (phosphatidylinositol-3-hydroxykinase) activation (51). From this evidence, we hypothesized that Dex-mediated antiviral effects depended on GR and NHE3 activity. To test this, we used RU486 (GR antagonist), tenapanor (specific NHE3 inhibitor), SGK1-IN-1 (selective SGK-1 inhibitor), and LY294002 (broad-spectrum PI3K inhibitor) as entry points to verify whether Dex antiviral effects relied on GR and NHE3 activity activation. *In vitro* cytotoxicity assays for RU486, tenapanor, and SGK1-IN-1 are shown (Fig. S3A through C). LY294002 cytotoxicity assays in DF-1 cells were performed according to a previous study (64). DF-1 cells were treated with LY294002, SGK1-IN-1, tenapanor, and RU486 for 2 h, then followed by IBV infection (MOI = 1). IBV replication was significantly inhibited by tenapanor (Fig. 6A), and notably, Dex-mediated antiviral effects were relieved by RU486 (Fig. 6B and C) and tenapanor (Fig. 6D and E).

We next determined tenapanor concentrations which antagonized Dex antiviral effects. In DF-1 cells, tenapanor concentrations of 5 µM and 1 µM were required to relieve Dex-mediated IBV inhibition (Fig. 6F and G). Similarly, in H1299 cells, a tenapanor concentration of 1 µM relieved Dex-mediated IBV inhibition (Fig. 6Hand I). Additionally, in DF-1 cells, tenapanor concentrations of 1 µM, 5 µM, and 10 µM allowed Dex to alleviate VSV inhibition. The results showed that the inhibitory effect of DEX on VSV was significantly weakened by tenapanor (Fig. 6J and K). Of note, IBV replication was only saved when the tenapanor concentration precisely counteracted Dex effects; otherwise, tenapanor continued to inhibit IBV replication. These results indicate that the Dex-mediated antiviral effects depend on GR and NHE3 activity.

### Dex and tenapanor significantly impact intracellular pH by regulating NHE3, which inhibits IBV replication

**Fig 7:**
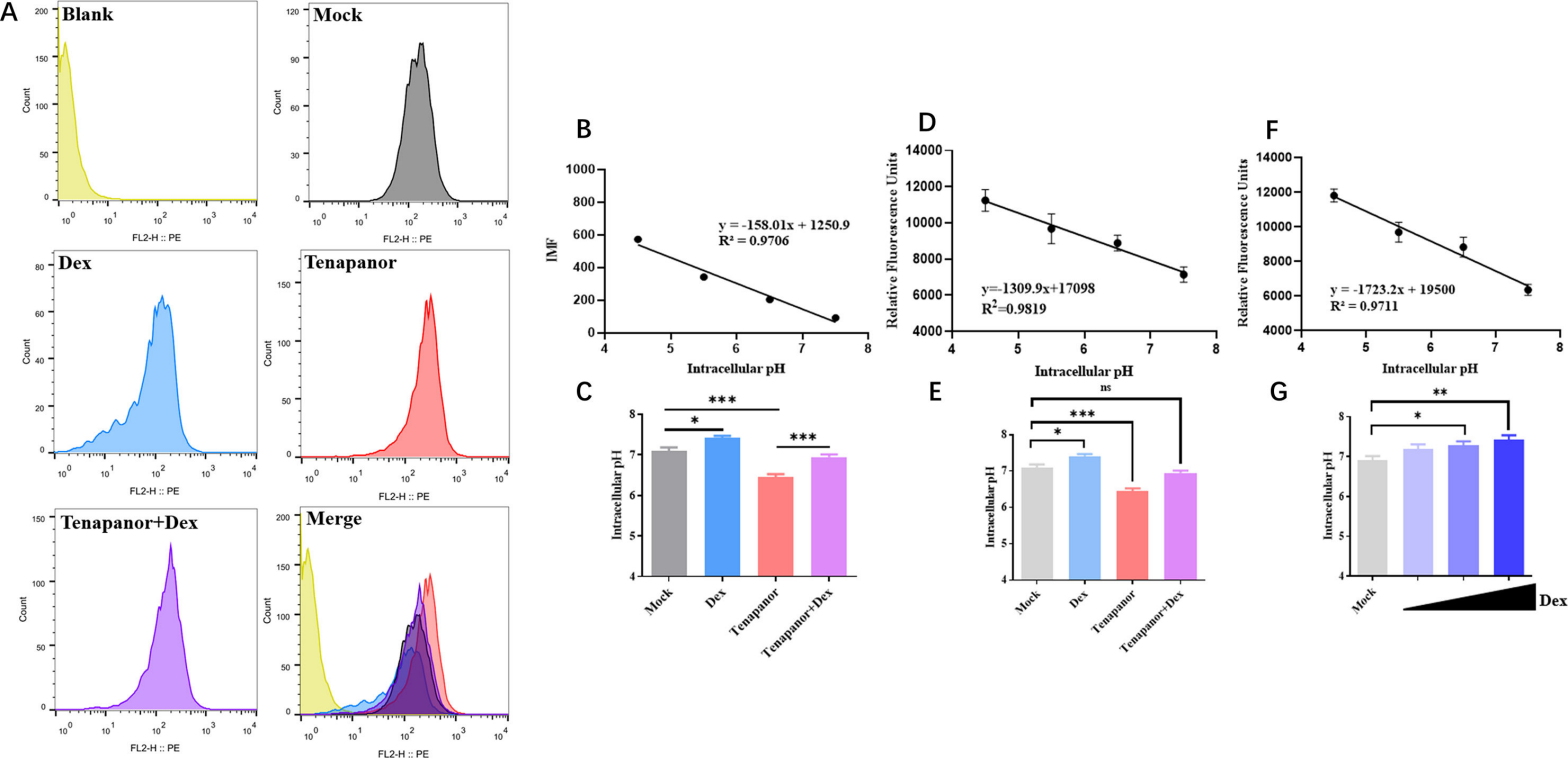
Dex and tenapanor effects on intracellular pH levels in DF-1 cells. DF-1 cells were treated with Dulbecco’s modified Eagle medium (DMEM) containing Dex (100 µg/mL) or tenapanor (10 µM). The experiment was divided into four groups: mock group (blank DMEM), Dex group (Dex treatment for 12 h), tenapanor group (tenapanor treatment for 2 h), tenapanor + Dex group (tenapanor treatment for 2 h first, followed by Dex treatment for 12 h). (**A**) Intracellular pH in DF-1 cells was determined by flow cytometry and pHrodo Red. (**B**) A standard curve was established during flow cytometry to determine a linear relationship between intracellular pH and the average pHrodo Red fluorescence intensity. (**C**) Histograms show intracellular pH quantification in DF-1 cells following Dex and tenapanor treatment (flow cytometry). (**D**) A standard curve was created using fluorescence microplate data to determine a linear relationship between intracellular pH and average pHrodo Red fluorescence intensity. (**E**) Histograms illustrate the quantification of intracellular pH in DF-1 cells following treatment with Dex and tenapanor, as determined by fluorescence microplate reader. (**E**) Histograms show intracellular pH quantification using mean fluorescence intensity (microplate reader). (**F**) A standard curve was created using fluorescence microplate reader data to determine a linear relationship between intracellular pH and average pHrodo Red fluorescence intensity. (**G**) Histograms show intracellular pH quantification in DF-1 cells following Dex (0 μg/mL–100 μg/mL) treatment for 24 h as determined by a fluorescence microplate reader.

Previous studies reported that cell surface NHE3 activity was regulated by Dex (50, 65, 66). We examined the impact of Dex and tenapanor on NHE3 activity in cell membranes using patch clamp technology and 2′, 7′-bis-(2-carboxyethyl)-5-(and-6)-carboxyfluorescein, acetoxymethyl ester mixed isomers (BCECF-AM) probes. Patch clamp results demonstrated similar changes in K^+^ and Na^+^ current density (Fig. S4A and S4B), with significant increases after Dex treatment and no notable alterations following tenapanor treatment. BCECF-AM probes revealed significant NHE3 activation due to Dex treatment, while tenapanor treatment substantially decreased this activity (Fig. 4C). Consequently, this generated a contrasting trend in intracellular pH between Dex and tenapanor treatments, which were putatively attributed to variations in H^+^ efflux rates resulting from differences in NHE3 activity (Fig. S4D). From these findings, we hypothesized that Dex and tenapanor modulated cellular pH homeostasis by regulating NHE3 activity, thereby suppressing IBV replication. To this end, intracellular pH was examined by flow cytometry and fluorescence microplate assays using pHrodo red AM probes. Flow cytometry indicated that Dex-treated cells showed increased intracellular pH when compared to mock cells (Fig. 7A through C). Conversely, tenapanor-treated cells showed decreased intracellular pH (Fig. 7A through C). However, tenapanor + Dex-treated cells showed intracellular pH levels similar to mock cells (Fig. 7A through C). Fluorescence microplate assay data were also consistent with flow cytometry data (Fig. 7D and E). Furthermore, fluorescence microplate assay data indicated a dose-dependent relationship between Dex and upregulation of intracellular pH in DF-1 cells after 24 h of treatment with different Dex concentrations (Fig. 7F and G).

While Dex appeared to have significant inhibitory effects toward IBV replication in DF-1 cells (Fig. 1A through D), effects in Vero cells were limited (Fig. 1F). Therefore, we examined Dex-mediated pH effects in these cells. As expected, no significant changes in pH levels were observed in Vero cells following Dex treatment (Fig. S5). This confirmed our hypothesis that Dex could not inhibit IBV replication in Vero cells due to unchanged pH. These data indicated that Dex and Tenapanor modulated intracellular pH homeostasis in DF-1 cells by influencing NHE3 activity, which inhibited IBV replication.

### IBV cell entry is significantly affected by pH homeostasis

**Fig 8:**
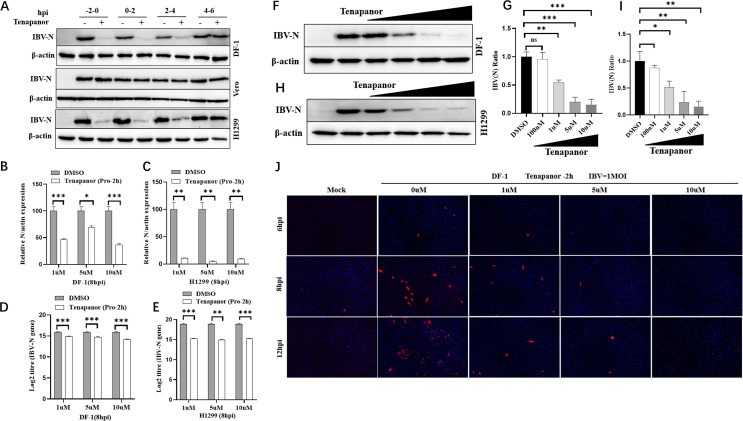
Tenapanor-induced pH homeostasis disruption affects early IBV infection steps. (**A**) DF-1, Vero, and H1299 cells were infected with IBV (MOI = 1) and incubated with tenapanor (10 µM) at −2–0 hpi, 0–2 hpi, 2–4 hpi, or 4–6 hpi. Cells were collected at 8 hpi to determine IBV-N protein levels by western blotting. DF-1 (B) and H1299 (**C**) cells were pretreated with tenapanor (1 µM, 5 µM and 10 µM) for 2 h and then infected with IBV (MOI = 1). Cells were harvested at 8 hpi, and *IBV-N* mRNA levels were determined using qRT-PCR. DF-1 (D) and H1299 (**E**) cells were pretreated with tenapanor (10 µM) for 2 h and then infected with IBV (MOI = 1). Cells were harvested at 8 hpi, and *IBV-N* mRNA levels were determined using absolute qRT-PCR. DF-1 (**F**) and H1299 (**H**) cells were incubated with increasing tenapanor (0 μM–10 μM) concentrations for 2 h, after which cells were infected with IBV (MOI = 1). Cells were collected at 8 hpi to determine IBV-N protein levels by western blotting. ImageJ software was used for the grayscale scanning of IBV-N protein bands in DF-1 (**G**) and H1299 (**I**) cells. (**J**) DF-1 cells were incubated with increasing tenapanor (0 μM–10 μM) concentrations for 2 h. Cells were then infected with IBV (MOI = 1). Immunofluorescence was performed at 6, 8, and 12 hpi using anti-IBV-N antibodies.

We previously suggested that IBV cell entry depended on low pH levels (62). However, intracellular pH homeostasis effects on early IBV infection steps are unclear. To investigate this specific IBV infection stage where pH was implicated, tenapanor was used to disrupt intracellular pH at various IBV infection stages. IBV-permissive DF-1, Vero, and H1299 cells were treated with 10 µM tenapanor for 2 h at pre-, during-, or post-IBV infection times at −2, 0, 2, and 4 hpi. The impact of intracellular pH on IBV infection was determined by examining IBV-N protein and IBV-N gene mRNA at 8 hpi. As shown (Fig. 8A), DF-1 and H1299 treatment with tenapanor at −2, 0, and 2 hpi blocked maximum IBV-N protein production. However, in Vero cells, tenapanor treatment at −2, 0, 2, and 4 hpi did not affect IBV-N protein production. Moreover, tenapanor significantly inhibited *IBV-N* mRNA levels in DF-1 and H1299 cells (Fig. 8B through E) and inhibited IBV replication in a dose-dependent manner (Fig. 8F through J). Based on earlier findings, we observed that IBV cell entry occurred within approximately 0–3 hpi (Fig. 3A), and at 3–4 hpi, IBV began to uncoat and produce IBV negative-strand genomic RNA (Fig. 3B). These observations further confirmed that tenapanor addition before IBV uncoating significantly inhibited IBV replication. Thus, intracellular pH homeostasis was essential for IBV replication, which mainly involved IBV entry steps.

### NHE3 is essential for IBV replication, which affects S protein cleavage

**Fig 9:**
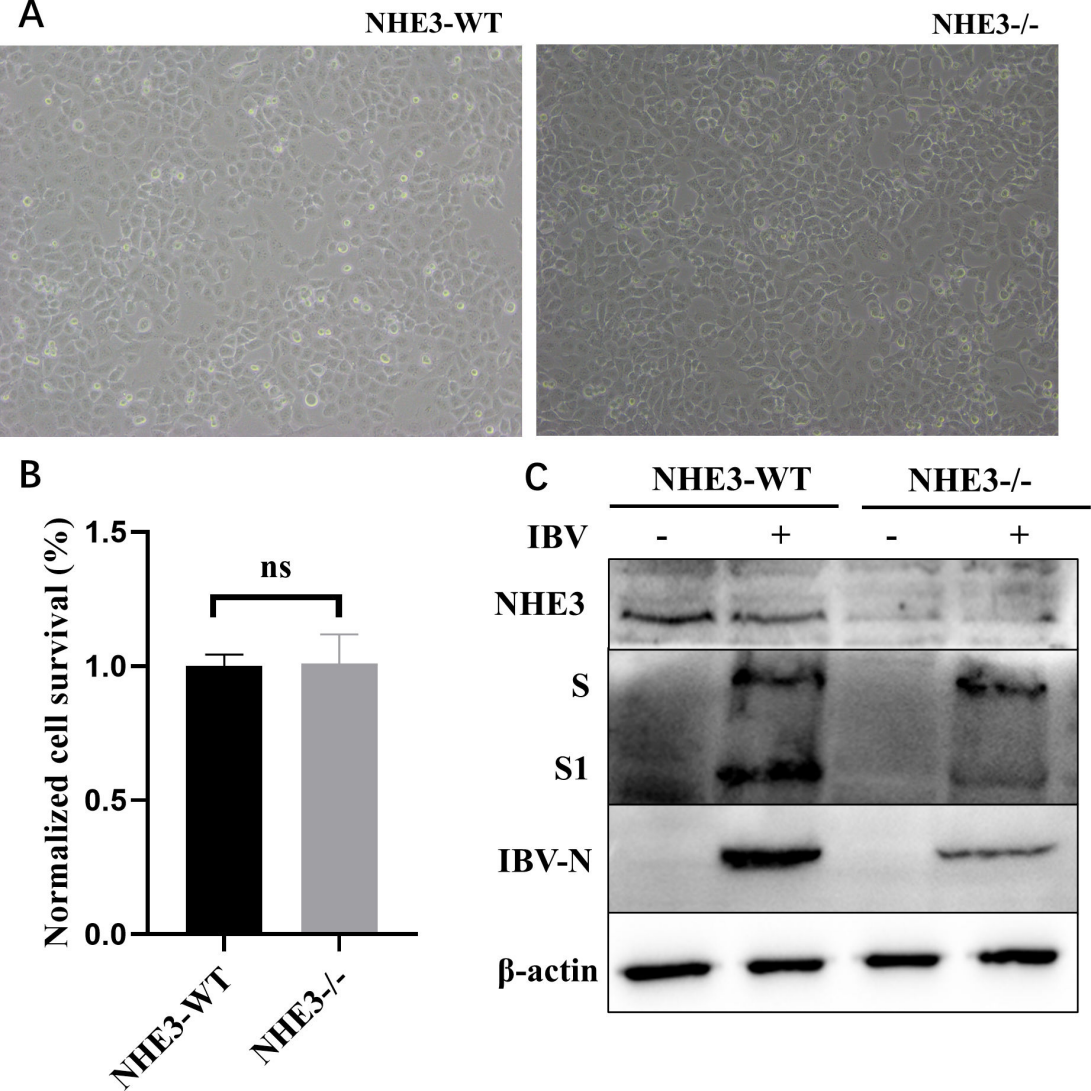
NHE3 is essential for IBV replication, which affects S protein cleavage. (**A**) NHE3-WT or NHE3^-/-^ H1299 cells were transfected with 3×Flag-NHE3, 3×Flag-NHE3-S663A, and 3×Flag-NHE3-S663D plasmids. At 24 h post-transfection, cells were infected with IBV (MOI = 1), after which cells were collected at 6 hpi for western blotting. (B, C) NHE3-WT or NHE3^-/-^ of H1299 cells were transfected with 3×Flag-NHE3, 3×Flag-NHE3-S663A, and 3×Flag-NHE3-S663D plasmids. At 24 h post-transfection, cells were infected with H9N2 (MOI = 1), after which cells were collected at 6 hpi for western blotting.

The NHE3^−/−^ H1299 cell line was created by knocking out the *SLC9A3* gene using CRISPR-Cas9-mediated genome editing technology (Fig. S6). In the cell counting kit-8 (CCK-8) assay, no statistically significant disparity in cell viability was observed between NHE3-wild-type (WT) and NHE3^-/-^ cells (Fig. 9A and B). Both cell lines were infected with IBV (MOI = 1). IBV-N protein levels were significantly inhibited in NHE3^-/-^ cells, while S protein cleavage was also inhibited (Fig. 9C). Therefore, NHE3 was essential for IBV replication, which affected S protein cleavage.

### Ser663 phosphorylation in NHE3 affects IBV replication

**Fig 10:**
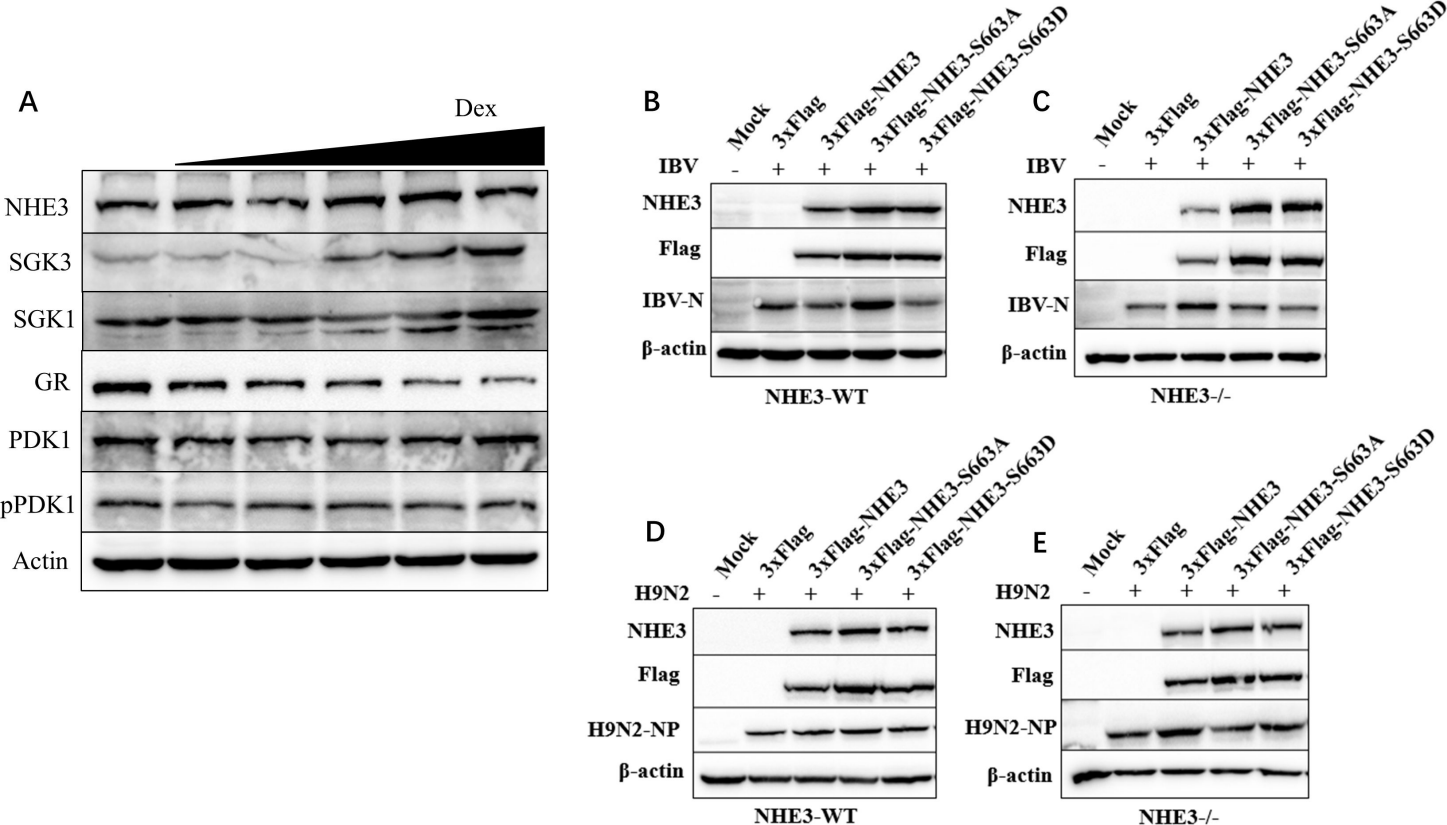
NHE3 phosphorylation at Ser663 inhibits IBV replication. (A) H1299 cells were pretreated with Dex (10 μg/mL) for 24 h, and then cells were collected to determine protein levels by western blotting. (B, C) NHE3-WT or NHE3^-/-^ H1299 cells were transfected with 3′Flag-NHE3, 3′Flag-NHE3-S663A, and 3′Flag-NHE3-S663D plasmids. At 24 h post-transfection, cells were infected with IBV at an MOI of 1, after which cells were collected at 6 hpi for western blotting. (D, E) NHE3-WT or NHE3^-/-^ H1299 cells were transfected with 3′Flag-NHE3, 3′Flag-NHE3-S663A, and 3′Flag-NHE3-S663D plasmids. At 24 h post-transfection, cells were infected with H9N2 at an MOI of 1, after which cells were collected at 6 hpi for western blotting.

Previous studies indicated that NHE3 activation by Dex requires NHE3 phosphorylation at Ser663 by SGK1 and SGK3, and also a functional GR (50, 51). Moreover, SGK3-mediated NHE3 activation was also dependent on PI3K and phosphoinositide-dependent kinase 1 (PDK1) (66). We observed that Dex did not alter SGK1 protein abundance in H1299 cells, consistent with previous studies (51). However, significantly upregulated SGK3 protein abundance was identified, while GR protein abundance was significantly downregulated. Interestingly, the abundance of NHE3 and PDK1 protein abundance remained unaffected by Dex (Fig. 10A). To investigate the impact of NHE3 phosphorylation on IBV replication, a mutant plasmid targeting NHE3 Ser663 was generated. Ser663 was mutated to alanine (Ala, A) to mimic dephosphorylation (NHE3-S663A), or to aspartate (Asp, D) to mimic phosphorylation (NHE3-S663D). Plasmids were transfected into H1299 cells which were grown for 24 h before infecting them with IBV (MOI = 1). After 6 hpi, samples were collected and analyzed for IBV-N protein levels by western blotting. In NHE3-WT cells, NHE3 dephosphorylation at Ser663 promoted IBV replication, while phosphorylation inhibited this process (Fig. 10B). In NHE3^-/-^ cells, IBV replication was unaffected by NHE3 phosphorylation (Fig. 10C). Additionally, phosphorylation and dephosphorylation at NHE3 Ser66 had almost no effects on H9N2 replication (Fig. 10D and E). Therefore, NHE3 phosphorylation at Ser663 affected IBV replication.

### Downregulated intracellular pH promotes avian influenza virus (AIV) replication

**Fig 11:**
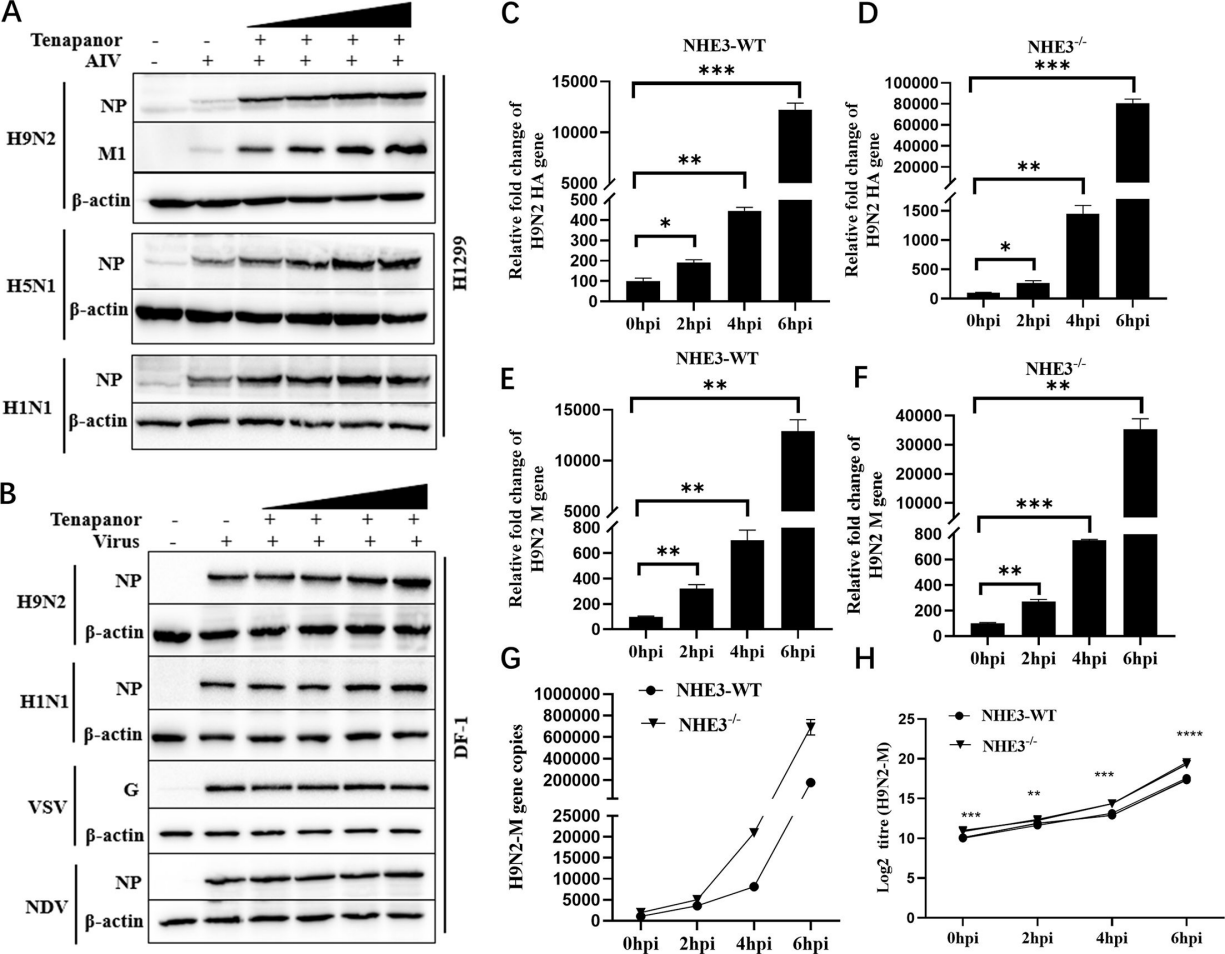
NHE3 activity affects influenza virus replication. (**A**) H1299 cells were pretreated with tenapanor (10 µM) for 2 h and then infected with H9N2, H5N1, and H1N1 (MOI = 1). Cells were collected at 6 hpi to determine virus protein levels by western blotting. (**B**) DF-1 cells were pretreated with tenapanor (10 µM) for 2 h and then infected with H9N2, H1N1, VSV, and NDV (MOI = 1). Cells were harvested at 6 hpi to determine viral protein levels by western blotting. NHE3-WT or NHE3^-/-^ H1299 cells were infected with H9N2 (MOI = 1). Subsequently, qRT-PCR was used to determine *H9N2-HA* (**C, D**) and *M* mRNA levels (**E, F**). Absolute qPCR was also performed to determine *H9N2-M* copies (**G**) and RNA levels (**H**).

**Fig 12:**
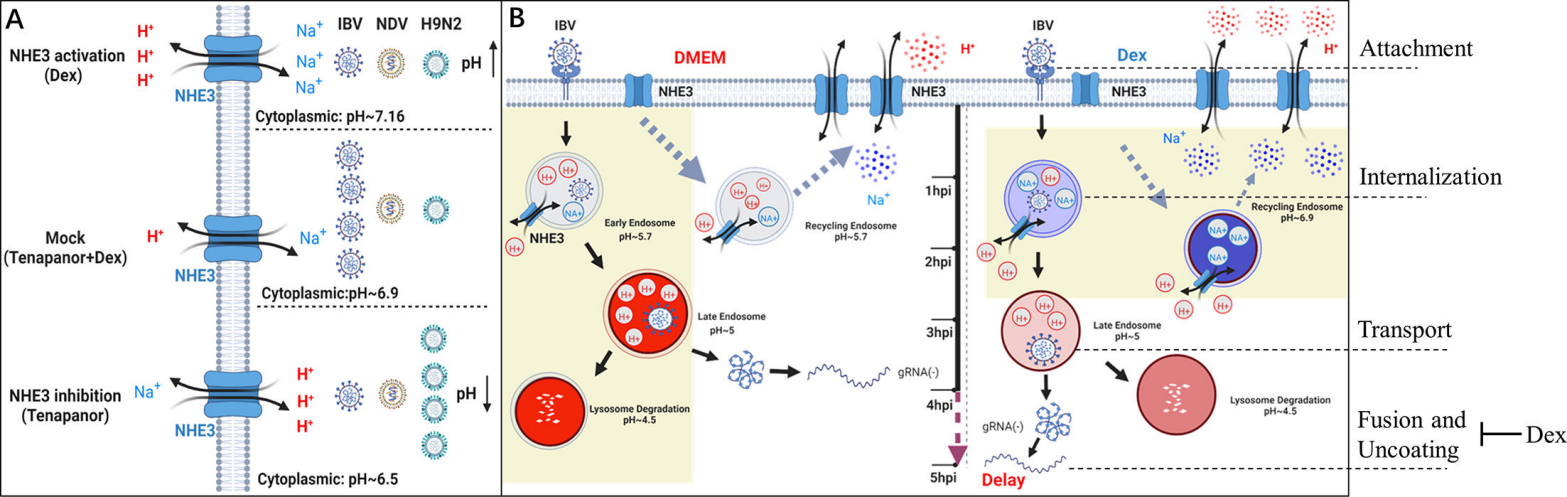
A working model showing how Dex activates NHE3 to inhibit IBV cell entry. (**A**) An intracellular acidic environment (pH) is required for IBV, NDV, and H9N2 virus replication. The optimal pH range for IBV replication is significantly narrow, whereas H9N2 prefers replication in acidic cytoplasmic environments. However, NDV replication has a high degree of adaptability to intracellular pH fluctuations. (**B**) Dex disrupts intracellular pH homeostasis to delay coronavirus IBV entry by activating NHE3.

Both Dex and tenapanor strongly inhibited IBV replication by affecting intracellular pH homeostasis induced by NHE3 activity (Fig. 1 and 8). To verify the impact of NHE3 activity on the replication of other viruses (AIV, NDV, and VSV), DF-1 and H1299 cells were exposed to varying tenapanor concentrations for 2 h. Cells were then infected with virus (MOI = 1). After 6 hpi, samples were collected, and viral protein levels were determined by western blotting. Tenapanor significantly enhanced AIV replication in H1299 cells, but the most prominent enhancement was observed for H9N2 replication (Fig. 11A). Moreover, in DF-1 cells, tenapanor also promoted H9N2 replication but had minimal effects on VSV and NDV replication (Fig. 11B). To examine NHE3 contributions to H9N2 replication in infected cells, NHE3-WT or NHE3^-/-^ H1299 cells were infected with H9N2 (MOI = 1). Samples were collected at indicated times and underwent *HA* and *M* gene RNA analyses by qPCR. We observed a noteworthy elevation in viral HA mRNA levels in NHE3^-/-^ cells, in direct contrast to NHE3-WT cells (Fig. 11C and D). Similar data were recorded for the *M* gene (Fig. 11E through H). These results demonstrate substantial discrepancies in intracellular acidic environmental (pH) requirements for IBV, NDV, and H9N2 replication. The optimum pH range for IBV replication was considerably narrow, whereas H9N2 exhibited a replication preference in acidic cytoplasmic environments (Fig. 12).

## DISCUSSION

Glucocorticoids are often used in combination with antiviral drugs to counteract painful inflammation and inhibit viral replication (67, 68). During the COVID-19 outbreak, a randomized, controlled clinical trial in the UK was the first to show that Dex reduced deaths and saved the lives of seriously ill patients with COVID-19 (69–73). However, controversy has ensued regarding glucocorticoid use for coronavirus treatment (74), including issues with efficacy, appropriate initiation times, dose, and treatment duration (75–77). However, some studies have reported that Dex treatment may increase viral yields and susceptibility, thereby increasing lung lesions (78–88). Consequently, glucocorticoid use is often hampered by adverse effects or resistance (89), meaning that glucocorticoids are a double-edged sword in the clinic. In this study, we identified a mechanism whereby glucocorticoids counteracted coronaviruses in cell models, which lays the foundation for novel drug development.

Coronavirus cell entry is key for virus replication cycles and evading host antiviral responses (1, 2). Currently, it is widely accepted that coronavirus cell entry occurs via the endocytic pathway, then passage through endosomal compartments via multivesicular bodies, and finally entry into the cytoplasm (3, 15, 62, 90, 91). In the 1930s, IBV was the first identified coronavirus which infected avian species and belonged to the genus *Gammacoronavirus* of the family *Coronaviridae* (92). Our previous studies demonstrated that low pH levels in intracytoplasmic vesicles were crucial for IBV entry (62). Moreover, IBV particles were transported via early and late endosomes and fused with late endosome-lysosome membranes to release nucleocapsids (62). Importantly, we also previously confirmed that IBV membrane fusion occurred from 2.5 to 3 hpi, and that IBV genome and related protein escape times from late endosomes or lysosomes was approximately 3.5–4 hpi (93). After uncoating, coronavirus genomic RNA separates from N protein to initiate negative-strand genomic RNA and virus genomic RNA synthesis. By examining *IBV-N* mRNA levels from 0 to 6 hpi, we observed that these levels were markedly suppressed by Dex after 3.5 hpi (Fig. 3A). Additionally, Dex blocked IBV negative-strand genomic RNA synthesis (Fig. 3B and C), and Dex treatment did not affect IBV adsorption (0 hpi) in DF-1 and H1299 cells (Fig. S1). These observations indicated that the suppression of initial IBV negative-strand genomic RNA synthesis was possibly due to blocked membrane fusion or virus uncoating by Dex.

The pH of the cytoplasm or endosomes plays a crucial role in the membrane fusion and uncoating of the coronavirus (62). Coronavirus not only relies on endosome acidification to facilitate replication, but also uses low-pH environments for immune evasion (21, 23, 25, 26). We identified notable disparities in IBV replication suppression by Dex in DF-1, H1299, and Vero cells (Fig. 1F and G). These discrepancies were possibly due to variations in individual cell responses to Dex treatment or significant variations in cell pH regulation caused by this treatment. Specifically, Vero cells exhibited a minimal impact on intracellular pH levels following Dex treatment (Fig. S5), while DF-1 cells showed significant changes (Fig. 7). Additionally, we successfully identified similar results in cytoplasmic pH changes following DF-1 and Vero cells treatment with tenapanor (Fig. 7 and Fig. S5). These findings further emphasized that the cytoplasmic pH in Vero cells remained largely unaffected after Dex and tenapanor treatments, which potentially explained why IBV replication was not influenced by Dex and tenapanor.

In general, coronaviruses enter cells by endocytosis, which undergoes pH-dependent membrane fusion (20, 94). The membrane fusion occurs within late endosomes and lysosomes after the S protein is cleaved by a low-pH-dependent protease; after that, the nucleocapsid is released into the cytosol, and the viral genomic RNA is uncoated (93). Previous studies also reported that coronavirus IBV-induced membrane fusion occurred at near-neutral pH (95). In our study, this suggested that both Dex and tenapanor treatments in DF-1 and H1299 cells efficiently suppressed IBV replication by inducing intracellular pH alterations. Moreover, we identified significant disparities regarding the intracellular pH range required for NDV, IBV, and H9N2 replication. Specifically, the optimum pH range for IBV replication was considerably narrow, whereas H9N2 exhibited a replication preference in acidic cytoplasmic environments (Fig. 12). Previous studies have also reported that influenza viruses enter the host cells through receptor-mediated endocytosis. Triggered by the decrease in pH in late endosomes during virus entry, HA undergoes conformational changes that underlie its transition from a prefusion state to a postfusion state (96–98). Therefore, both the coronavirus and the influenza virus require a suitable intracellular pH environment to enter cells.

NHE3 has crucial roles in receptor-mediated endocytosis, contributing to endosomal acidification and maintaining cytoplasmic pH homeostasis (39–41). Inhibiting NHE3 activity causes early endosomal alkalinization and cytoplasmic acidification, making NHE3 a significant determinant in cellular processes (28). Currently, little is known about the connections between IBV replication and NHE3 activity. To this end, our data indicated that Dex inhibited IBV replication by regulating NHE3 activity via the GR, which affected cytoplasmic or intracytoplasmic vesicle pH (Fig. 4, 6, and 7). Also, cytoplasmic pH homeostasis was required for IBV cell entry when tenapanor was supplemented to cells between −2 and 0 hpi, 0 and 2 hpi, 2 and 4 hpi, and 4 and 6 hpi, suggesting that NHE3 activity was required for early infection stages (Fig. 8A). Moreover, NHE3 dephosphorylation at Ser663 promoted IBV replication, while phosphorylation inhibited this process (Fig. 10B). Other studies have also reported crucial NHE3 activity roles in the pathogenicity of other coronaviruses (44–48). For instance, the diminished activity of the intestinal surface Na^+^/H^+^ exchanger NHE3 stands as a pivotal factor in precipitating diarrhea following PEDV infection in neonatal piglets (45). Specifically, the decrease in NHE3 functionality within intestinal epithelial cells, a common occurrence in both TGEV- and PEDV-induced diarrhea among piglets (44), underscores the mechanism whereby PEDV impedes Na^+^ transportation by suppressing NHE3 activity, ultimately resulting in diarrhea (45). Furthermore, TGEV infection exerts its influence by inhibiting NHE3 translocation and attenuating sodium hydrogen exchange activity through the intricate SGLT1-mediated p38 mitogen-activated protein kinase (MAPK)/Akt2 signaling cascade. This interference disrupts cellular electrolyte absorption, a crucial process, thereby contributing to the onset of diarrhea (46). Intriguingly, in TGEV-infected intestinal epithelial cells, epidermal growth factor receptor (EGFR) emerges as a negative regulator of NHE3 activity (47), highlighting another layer of complexity in the pathogenesis. Additionally, TGEV N protein exerts a direct influence on NHE3 expression and activity via protein-protein interactions, a vital step in facilitating the development of diarrhea (99). Therefore, we hypothesize that targeted NHE3 interference using drugs may impact coronavirus pathogenicity (27).

Previous studies have highlighted the important role of endosomal pH in the pathogenicity of coronaviruses (2, 5, 6, 20, 21). In the study, we delved into the mechanism by which Dex disrupts intracellular pH homeostasis, thereby delaying the entry of the IBV into cells through the activation of NHE3. While this observation represents a significant cellular-level finding that Dex has a potent inhibitory effect on IBV, our prior research has shown that Dex can actually promote IBV replication *in vivo*, likely due to the immunosuppressive effects induced by the drug (100).

Glucocorticoids, such as Dex, have been extensively studied both *in vivo* and *in vitro* with various viruses, revealing a spectrum of effects that can be quite divergent. For instance, glucocorticoid treatment has been shown to significantly reduce mucus production in respiratory syncytial virus (RSV)-infected cells, yet paradoxically increases RSV viral load both *in vitro* and *in vivo* (6). Similarly, glucocorticoid treatment has been associated with an enhancement of human metapneumovirus replication (101). Furthermore, Dex-induced immunosuppression has been found to affect the viral RNA levels of the Zika virus in the blood and multiple tissues of infected mice (102). In the context of COVID-19 patients, Dex has been reported to mitigate cytokine storms (55, 103) and improve survival rates in critically ill patients (77). These findings suggest that the molecular mechanisms underlying Dex’s dual role in either promoting or inhibiting viral replication are exceptionally complex, likely involving multiple factors such as cellular pH, cytokine storms, immune suppression, and cellular mucus production. Consequently, further research is imperative to elucidate how to harness its antiviral potential without compromising immune function. In this study, we discovered that Dex disrupts intracellular pH homeostasis to delay IBV cell entry via NHE3 activation. Targeting NHE3 activity without compromising immune function could potentially emerge as a novel strategy for developing anti-coronavirus therapeutics. These findings may serve as a valuable reference for the development of anti-coronavirus drugs, especially in the context of targeting NHE3 activity—a strategy that could offer a novel approach to the development of antiviral agents without compromising immune health.

### Conclusions

In summary, our results provide new information that NHE3 activity is crucial for intracellular pH homeostasis, which is essential for coronavirus membrane fusion and uncoating. Therefore, the development of selective pH-regulating drugs that target NHE3 activity to enhance the proton leakage pathway between the plasma membrane and endosomes could potentially serve as an effective strategy to combat early coronavirus infection.

## MATERIALS AND METHODS

### Cells and viruses

DF-1, H1299, Vero, HeLa, and A549 cells were obtained from the American Type Culture Collection (ATCC) (62, 104). Porcine kidney epithelial cells (PK1) were kindly provided by Professor Tongling Shan (Shanghai Veterinary Research Institute, Chinese Academy of Agricultural Sciences). DF-1, H1299, Vero, HeLa, A549, and PK1 cell culture was performed according to our previous methods (62, 104–107). Briefly, DF-1 and Vero cells were grown in Dulbecco’s Modified Eagle Medium (Gibco, C11995500BT) plus 10% fetal bovine serum (FBS; Gibco, 10270106). H1299 and A549 cells were maintained in Roswell Park Memorial Institute 1640 medium plus 10% FBS. PK1 cells were maintained in Minimum Eagle’s Medium plus 10% FBS. All cells were cultured at 37°C under 5% CO_2_. IBV (ATCC VR-22), VSV, AIV, and NDV strains were kept at the Shanghai Veterinary Research Institute (CAAS, Shanghai) (104, 108, 109). PEDV (HLJBY strain) was kindly provided by Professor Mao Xiang (93). The IBV Beaudette strain used in this study was adapted to DF-1 cells and was a gift from Professor Dingxiang Liu (South China Agricultural University) (62, 108).

### Chemical reagents

The intracellular pH calibration buffer Kit (cat. no. P35379), live cell imaging solution (A14291DJ), and pHrodo Red AM (P35372) were purchased from Thermo Fisher Scientific. The NHE3-specific inhibitor tenapanor (HY-15991), PI3K inhibitor LY294002 (HY-10108), SGK-1 inhibitor SGK1-IN-1 (HY-18607), and GR antagonist RU486 (HY-13683) were purchased from MedChemExpress. BCECF-AM (CS314) was purchased from ZoFtic. SYBR green qPCR master mix (11184ES08) was purchased from Yeasen Biotechnology Co., Ltd. (Shanghai). Protonex Red 600 (AAT-B21207) was purchased from AAT Bioquest.

### Western blotting and antibodies

Cells were harvested at indicated infection times, lysed in 2×  sodium dodecyl sulfate (SDS) loading buffer plus 100 mM dithiothreitol, and denatured at 100°C for 10 min. Protein samples were separated according to a previous method (104). In short, identical quantities of protein were effectively separated through SDS-PAGE, and subsequently transferred onto polyvinylidene fluoride (PVDF) membranes utilizing the electroblotting method. Immunoblot analysis was then performed by incubating membranes with blocking buffer for 1 h at room temperature and incubating with appropriate antibodies diluted in blocking buffer for 1 h. Following three washes with phosphate-buffered saline with Tween-20 (PBST), the membranes were incubated with an horseradish peroxidase (HRP)-conjugated secondary antibody for 1 h, followed by three additional washes with PBST. The blots were then developed using an advanced enhanced chemiluminescence detection system and exposed to a chemiluminescence gel imaging system (Tanon 5200, Tanon, Guangzhou, China) for visualization.

The following antibodies were used: anti-IBV-N, anti-IBV-nsp3, and anti-IBV-nsp15 polyclonal antibodies were obtained by immunizing rabbits with respective antigens in our laboratory (93, 104). Mouse monoclonal anti-IBV-S1 and anti-IBV-S2 antibodies were gifts from Professor Min Liao (Zhejiang University, China). The anti-PEDV N antibody was a gift from Professor Zhou Yanjun (Shanghai Veterinary Research Institute, Shanghai, China). Mouse monoclonal anti-NDV-NP antibody was prepared in our laboratory (110). β-Actin (66009-1-lg), NHE3 (27190-1-AP), SGK3 (12699-1-AP), SGK1 (28454-1-AP), and PDK1 (29241-1-AP) antibodies came from Proteintec. H1N1-NP (A01506) came from GenScript Biotech; VSV-G (ab1874) came from Abcam; HSV1 (NB600-516) came from Novus Biologicals; ATP6AP1 (sc-515607) came from Santa Cruz Biotechnology; IAV-M1 (GTX125928) came from GeneTex; and Rab5 (#3547), Rab7 (#9367), GR (#12041), and LAMP1 (#9091) came from Cell Signaling Technology. HRP-conjugated goat anti-mouse IgG (SA00001-1) and HRP-conjugated goat anti-rabbit IgG (SA00001-2) were purchased from Proteintech. Alexa Fluor Plus 488-conjugated goat anti-mouse IgG (A32723) and Alexa Fluor 594-conjugated goat anti-rabbit IgG (A32740) were purchased from Invitrogen.

### Immunofluorescence

DF-1 and H1299 cells were seeded on cover slips in six-well plates and infected with the IBV Beaudette strain. Cells were harvested at indicated times and fixed in 4% paraformaldehyde for 30 min. After three washes in phosphate-buffered saline (PBS), cells were permeabilized in 0.1% Triton X-100 (Merck, 9002-93-1) in PBS for 15 min and blocked in 5% bovine serum albumin (Beijing ZEPING Bioscience & Technology, 02FC007780) in PBS. Cells were then incubated overnight with primary antibodies (1:200) at 4°C. The next day, cells were washed in Tris-buffered saline and Tween-20 (TBST) and incubated with an Alexa Fluor-conjugated secondary antibody for 1 h at 37°C. Next, 4',6-diamidino-2-phenylindole (DAPI) was applied for 15 min to stain nuclei. Following this, cells were washed once in TBST and examined under a confocal microscope (Zeiss LSM880O). Finally, images were analyzed using ImageJ software.

### RNA isolation and RT-qPCR

RNA was extracted using an RNeasy Mini Kit (QIAGEN, ID: 74104, Germany) according to manufacturer’s protocols. The qRT-PCR assay was performed as described earlier (111). IBV, H9N2, and NDV RNAs were detected according to a qRT-PCR assay established in our laboratory (100, 112). The qRT-PCR standard curve equation for the IBV *N* gene was *y* = −3.287x + 39.25 (*R*^2^ = 0.993). IBV negative genomic RNA detection methods were as previously described (61). The qRT-PCR standard curve equation for the H9N2 virus *M* gene was *y* = 35.619 − 3.321x (*R*^2^ = 0.999). qRT-PCR parameters were as follows: 94°C for 5 min, and then 40 cycles at 94°C for 15 s, annealing at 59°C for 15 s, and extension at 72°C for 15 s. NDV detection methods were as previously described (113). All qRT-PCR primers are listed in Table S1.

### Cytoplasmic pH detection using pHrodo Red AM

The pHrodo Red AM (P35372, Thermo Fisher Scientific) intracellular pH indicator was specifically designed to measure cytosolic pH. A standard curve was created using pHrodo Red AM and an intracellular pH calibration buffer kit (cat. no. P35379, Thermo Fisher Scientific). The kit contains a range of pH calibration buffers at pH 4.5, 5.5, 6.5, and 7.5, as well as valamycin and nigericin to help balance the pH inside and outside the cell. Absolute pH values were calculated by converting arbitrary pH ratios (from light excited at 560 nm and emitted at 580 nm) using an intracellular pH calibration curve kit (114).

NHE activity measurements

Intracellular pH changes are attributed to NHE activation (115). BCECF-AM is a cell membrane-permeable compound widely used as a fluorescence indicator of intracellular pH (115, 116). We used this indicator to measure cytosolic pH and NHE activity changes induced by Dex and tenapanor. In short, cells were treated with Dex and tenapanor to prepare a cell density of approximately 1 × 10^6^/mL. Then, BCECF-AM storage solution (1 mM) was diluted 100–500 times in PBS. Then, the diluted solution was mixed thoroughly with the cell suspension in a 1:1 volume ratio, mixed well, and incubated at 4°C or 37°C for 15 min–60 min, and cells were finally washed in fresh cell culture medium. Once the fluorescent probe had passed through cell membranes, cells were suspended in a sodium-free solution, and NaCl (130 mM) was added. At this point, the majority of intracellular pH changes had originated from NHE exchange. Finally, a fluorescence or laser confocal microscope with an image analysis system was used to detect fluorescence intensity in cells.

### Cell viability assay

Cell viability was determined by CCK-8 as previously described (112).

### Statistical analysis

The data presented in this study represent the means of at least three independent experiments. Statistical analysis was conducted by GraphPad Prism software 9.0 (GraphPad Software, Inc.). All data are expressed as means ± standard error of the mean. Statistical significance was determined using an unpaired Student’s *t*-test. A *P*-value of <0.05 was considered statistically significant. The notation used to indicate significance is as follows: ns for *P* > 0.05, * for *P* < 0.05, ** for *P* < 0.01, and *** for *P* < 0.001.

## Data Availability

Sequences of all primers, details of the cloning, and plasmids used in this study are available upon request.

## SUPPLEMENTAL MATERIAL

Figure S1 jvi.01894-24-s0001.docx qRT-PCR was performed to evaluate the effects of Dex on IBV.

Figure S2 jvi.01894-24-s0002.docx Protonex Red 600 pH fluorescent probe was performed to evaluate the effects of Dex on pH in endo-lysosomes.

Figure S3 jvi.01894-24-s0003.docx CCK-8 was used to evaluate the cell activity.

Figure S4 jvi.01894-24-s0004.docx Dex effects on Na+/H+ exchange in H1299 cell membranes.

Figure S5 jvi.01894-24-s0005.docx Dex and Tenapanor effects on intracellular pH levels in Vero cells.

Figure S6 jvi.01894-24-s0006.docx Construction and validation of SLC9A3 gene knockout cell line.

Table S1 jvi.01894-24-s0007.docx All qRT-PCR primers used in this study.

ASM does not own the copyrights to Supplemental Material that may be linked to, or accessed through, an article. The authors have granted ASM a non-exclusive, world-wide license to publish the Supplemental Material files. Please contact the corresponding author directly for reuse.

## References

[1] Taylor MP, Koyuncu OO, Enquist LW. 2011. Subversion of the actin cytoskeleton during viral infection. Nat Rev Microbiol 9:427–439. doi:10.1038/nrmicro2574 21522191 PMC3229036

[2] Hoffmann M, Kleine-Weber H, Schroeder S, Krüger N, Herrler T, Erichsen S, Schiergens TS, Herrler G, Wu NH, Nitsche A, Müller MA, Drosten C, Pöhlmann S. 2020. Sars-cov-2 cell entry depends on ace2 and tmprss2 and is blocked by a clinically proven protease inhibitor. Cell 181:271–280. doi:10.1016/j.cell.2020.02.052 32142651 PMC7102627

[3] Dai J, Wang H, Liao Y, Tan L, Sun Y, Song C, Liu W, Qiu X, Ding C. 2022. Coronavirus infection and cholesterol metabolism. Front Immunol 13:791267. doi:10.3389/fimmu.2022.791267 35529872 PMC9069556

[4] Wei C, Wan L, Zhang Y, Fan C, Zhong H. 2020. Cholesterol metabolism--impact for sars-cov-2 infection prognosis, entry, and antiviral therapies. MedRxiv

[5] Wang H, Yang P, Liu K, Guo F, Zhang Y, Zhang G, Jiang C. 2008. SARS coronavirus entry into host cells through a novel clathrin- and caveolae-independent endocytic pathway. Cell Res 18:290–301. doi:10.1038/cr.2008.15 18227861 PMC7091891

[6] Yang ZY, Huang Y, Ganesh L, Leung K, Kong WP, Schwartz O, Subbarao K, Nabel GJ. 2004. pH-dependent entry of severe acute respiratory syndrome coronavirus is mediated by the spike glycoprotein and enhanced by dendritic cell transfer through DC-SIGN. J Virol 78:5642–5650. doi:10.1128/JVI.78.11.5642-5650.2004 15140961 PMC415834

[7] Matsuyama S, Taguchi F. 2009. Two-step conformational changes in a coronavirus envelope glycoprotein mediated by receptor binding and proteolysis. J Virol 83:11133–11141. doi:10.1128/JVI.00959-09 19706706 PMC2772765

[8] Li X, Zhu W, Fan M, Zhang J, Peng Y, Huang F, Wang N, He L, Zhang L, Holmdahl R, Meng L, Lu S. 2021. Dependence of SARS-CoV-2 infection on cholesterol-rich lipid raft and endosomal acidification. Comput Struct Biotechnol J 19:1933–1943. doi:10.1016/j.csbj.2021.04.001 33850607 PMC8028701

[9] Bayati A, Kumar R, Francis V, McPherson PS. 2021. SARS-CoV-2 infects cells after viral entry via clathrin-mediated endocytosis. J Biol Chem 296:100306. doi:10.1016/j.jbc.2021.100306 33476648 PMC7816624

[10] Tang T, Jaimes JA, Bidon MK, Straus MR, Daniel S, Whittaker GR. 2021. Proteolytic activation of sars-cov-2 spike at the s1/s2 boundary: potential role of proteases beyond furin. ACS Infect Dis 7:264–272. doi:10.1021/acsinfecdis.0c00701 33432808

[11] Chu VC, McElroy LJ, Chu V, Bauman BE, Whittaker GR. 2006. The avian coronavirus infectious bronchitis virus undergoes direct low-pH-dependent fusion activation during entry into host cells. J Virol 80:3180–3188. doi:10.1128/JVI.80.7.3180-3188.2006 16537586 PMC1440383

[12] Fang P, Zhang J, Zhang H, Xia S, Ren J, Tian L, Bai D, Fang L, Xiao S. 2021. Porcine deltacoronavirus enters porcine IPI-2I intestinal epithelial cells via macropinocytosis and clathrin-mediated endocytosis dependent on pH and dynamin. J Virol 95:e0134521. doi:10.1128/JVI.01345-21 34586858 PMC8610596

[13] Li S, Xiao D, Zhao Y, Zhang L, Chen R, Liu W, Wen Y, Liao Y, Wen Y, Wu R, Han X, Zhao Q, Du S, Yan Q, Wen X, Cao S, Huang X. 2022. Porcine deltacoronavirus (PDCoV) entry into PK-15 cells by caveolae-mediated endocytosis. Viruses 14:496. doi:10.3390/v14030496 35336903 PMC8950576

[14] Li Z, Zhao K, Lan Y, Lv X, Hu S, Guan J, Lu H, Zhang J, Shi J, Yang Y, Song D, Gao F, He W. 2017. Porcine hemagglutinating encephalomyelitis virus enters Neuro-2a cells via clathrin-mediated endocytosis in a Rab5-, cholesterol-, and pH-dependent manner. J Virol 91:10-1128. doi:10.1128/JVI.01083-17 PMC5686734 28956766

[15] Wei X, She G, Wu T, Xue C, Cao Y. 2020. PEDV enters cells through clathrin-, caveolae-, and lipid raft-mediated endocytosis and traffics via the endo-/lysosome pathway. Vet Res 51:10. doi:10.1186/s13567-020-0739-7 32041637 PMC7011528

[16] Qiu Z, Hingley ST, Simmons G, Yu C, Das Sarma J, Bates P, Weiss SR. 2006. Endosomal proteolysis by cathepsins is necessary for murine coronavirus mouse hepatitis virus type 2 spike-mediated entry. J Virol 80:5768–5776. doi:10.1128/JVI.00442-06 16731916 PMC1472567

[17] Eifart P, Ludwig K, Böttcher C, de Haan CAM, Rottier PJM, Korte T, Herrmann A. 2007. Role of endocytosis and low pH in murine hepatitis virus strain A59 cell entry. J Virol 81:10758–10768. doi:10.1128/JVI.00725-07 17626088 PMC2045462

[18] Hansen GH, Delmas B, Besnardeau L, Vogel LK, Laude H, Sjöström H, Norén O. 1998. The coronavirus transmissible gastroenteritis virus causes infection after receptor-mediated endocytosis and acid-dependent fusion with an intracellular compartment. J Virol 72:527–534. doi:10.1128/JVI.72.1.527-534.1998 9420255 PMC109404

[19] Tanaka Y, Tanabe E, Nonaka Y, Uemura M, Tajima T, Ochiai K. 2022. Lonophore antibiotics inhibit type II feline coronavirus proliferation in vitro. Viruses 14:1734. doi:10.3390/v14081734 36016355 PMC9415497

[20] Liu T, Luo S, Libby P, Shi GP. 2020. Cathepsin L-selective inhibitors: a potentially promising treatment for COVID-19 patients. Pharmacol Ther 213:107587. doi:10.1016/j.pharmthera.2020.107587 32470470 PMC7255230

[21] Zhou T, Tsybovsky Y, Gorman J, Rapp M, Cerutti G, Chuang GY, Katsamba PS, Sampson JM, Schön A, Bimela J. 2020. Cryo-EM structures of sars-cov-2 spike without and with ace2 reveal a pH-dependent switch to mediate endosomal positioning of receptor-binding domains. Cell Host Microbe 28:867–879. doi:10.1016/j.chom.2020.11.004 33271067 PMC7670890

[22] Lv J, Wang Z, Qu Y, Zhu H, Zhu Q, Tong W, Bao L, Lv Q, Cong J, Li D, Deng W, Yu P, Song J, Tong WM, Liu J, Liu Y, Qin C, Huang B. 2021. Distinct uptake, amplification, and release of SARS-CoV-2 by M1 and M2 alveolar macrophages. Cell Discov 7:24. doi:10.1038/s41421-021-00258-1 33850112 PMC8043100

[23] Yang N, Shen HM. 2020. Targeting the endocytic pathway and autophagy process as a novel therapeutic strategy in COVID-19. Int J Biol Sci 16:1724–1731. doi:10.7150/ijbs.45498 32226290 PMC7098027

[24] Chen X, Geiger JD. 2020. Janus sword actions of chloroquine and hydroxychloroquine against COVID-19. Cell Signal 73:109706. doi:10.1016/j.cellsig.2020.109706 32629149 PMC7333634

[25] Ghosh S, Dellibovi-Ragheb TA, Kerviel A, Pak E, Qiu Q, Fisher M, Takvorian PM, Bleck C, Hsu VW, Fehr AR, Perlman S, Achar SR, Straus MR, Whittaker GR, de Haan CAM, Kehrl J, Altan-Bonnet G, Altan-Bonnet N. 2020. β-coronaviruses use lysosomes for egress instead of the biosynthetic secretory pathway. Cell 183:1520–1535. doi:10.1016/j.cell.2020.10.039 33157038 PMC7590812

[26] Hou Y, Wang T, Ding Y, Yu T, Cui Y, Nie H. 2022. Expression profiles of respiratory V-ATPase and calprotectin in SARS-CoV-2 infection. Cell Death Discov 8:362. doi:10.1038/s41420-022-01158-3 35974012 PMC9379883

[27] Prasad H. 2021. Protons to patients: targeting endosomal Na /H exchangers against COVID-19 and other viral diseases. FEBS J 288:5071–5088. doi:10.1111/febs.16163 34490733 PMC8646450

[28] Gekle M, Drumm K, Mildenberger S, Freudinger R, Gassner B, Silbernagl S. 1999. Inhibition of Na+-H+ exchange impairs receptor-mediated albumin endocytosis in renal proximal tubule-derived epithelial cells from opossum. J Physiol 520 Pt 3:709–721. doi:10.1111/j.1469-7793.1999.00709.x 10545138 PMC2269612

[29] Fukura N, Ohgaki R, Matsushita M, Nakamura N, Mitsui K, Kanazawa H. 2010. A membrane-proximal region in the C-terminal tail of NHE7 is required for its distribution in the trans-Golgi network, distinct from NHE6 localization at endosomes. J Membr Biol 234:149–158. doi:10.1007/s00232-010-9242-9 20364249

[30] Ohgaki R, van Ijzendoorn SCD, Matsushita M, Hoekstra D, Kanazawa H. 2011. Organellar Na+/H+ exchangers: novel players in organelle pH regulation and their emerging functions. Biochemistry-Us 50:443–450. doi:10.1021/bi101082e 21171650

[31] Ohgaki R, Matsushita M, Kanazawa H, Ogihara S, Hoekstra D, van Ijzendoorn SCD. 2010. The Na+/H+ exchanger NHE6 in the endosomal recycling system is involved in the development of apical bile canalicular surface domains in HepG2 cells. Mol Biol Cell 21:1293–1304. doi:10.1091/mbc.e09-09-0767 20130086 PMC2847532

[32] Cure MC, Cure E. 2022. Prolonged NHE activation may be both cause and outcome of cytokine release syndrome in COVID-19. Curr Pharm Des 28:1815–1822. doi:10.2174/1381612828666220713121741 35838211

[33] Pedersen SF, Counillon L. 2019. The SLC9A-C mammalian Na(+)/H(+) exchanger family: molecules, mechanisms, and physiology. Physiol Rev 99:2015–2113. doi:10.1152/physrev.00028.2018 31507243

[34] Donowitz M, Ming Tse C, Fuster D. 2013. SLC9/NHE gene family, a plasma membrane and organellar family of Na + SLC9/NHE gene family, a plasma membrane and organellar family of Na + SLC9/NHE gene family, a plasma membrane and organellar family of Na. Mol Aspects Med 34:236–251. doi:10.1016/j.mam.2012.05.001

[35] Ran L, Yan T, Zhang Y, Niu Z, Kan Z, Song Z. 2021. The recycling regulation of sodium-hydrogen exchanger isoform 3(NHE3) in epithelial cells. Cell Cycle 20:2565–2582. doi:10.1080/15384101.2021.2005274 34822321 PMC8726692

[36] Nwia SM, Li XC, Leite APdO, Hassan R, Zhuo JL. 2022. The Na^+^/H^+^ exchanger 3 in the intestines and the proximal tubule of the kidney: localization, physiological function, and key roles in angiotensin II-induced hypertension. Front Physiol 13:861659. doi:10.3389/fphys.2022.861659 35514347 PMC9062697

[37] He P, Yun CC. 2010. Mechanisms of the regulation of the intestinal Na+/H+ exchanger NHE3. J Biomed Biotechnol 2010:238080. doi:10.1155/2010/238080 20011065 PMC2789519

[38] Donowitz M, Li X. 2007. Regulatory binding partners and complexes of NHE3. Physiol Rev 87:825–872. doi:10.1152/physrev.00030.2006 17615390

[39] Kurashima K, Szabó EZ, Lukacs G, Orlowski J, Grinstein S. 1998. Endosomal recycling of the Na+/H+ exchanger NHE3 isoform is regulated by the phosphatidylinositol 3-kinase pathway. J Biol Chem 273:20828–20836. doi:10.1074/jbc.273.33.20828 9694828

[40] Janecki AJ, Montrose MH, Zimniak P, Zweibaum A, Tse CM, Khurana S, Donowitz M. 1998. Subcellular redistribution is involved in acute regulation of the brush border Na+/H+ exchanger isoform 3 in human colon adenocarcinoma cell line Caco-2. Protein kinase C-mediated inhibition of the exchanger. J Biol Chem 273:8790–8798. doi:10.1074/jbc.273.15.8790 9535857

[41] Graves AR, Curran PK, Smith CL, Mindell JA. 2008. The Cl-/H+ antiporter ClC-7 is the primary chloride permeation pathway in lysosomes. Nature 453:788–792. doi:10.1038/nature06907 18449189

[42] Prasad H. 2021. Protons to Patients: targeting endosomal Na(+) /H(+) exchangers against COVID-19 and other viral diseases. Febs j 288:5071–5088. doi:10.1111/febs.16163 34490733 PMC8646450

[43] Kondapalli KC, Llongueras JP, Capilla-González V, Prasad H, Hack A, Smith C, Guerrero-Cázares H, Quiñones-Hinojosa A, Rao R. 2015. A leak pathway for luminal protons in endosomes drives oncogenic signalling in glioblastoma. Nat Commun 6:6289. doi:10.1038/ncomms7289 25662504 PMC4354686

[44] Niu Z, Zhang Y, Kan Z, Ran L, Yan T, Xu S, Zhang S, Zhang J, Zou H, Song Z. 2021. Decreased NHE3 activity in intestinal epithelial cells in TGEV and PEDV-induced piglet diarrhea. Vet Microbiol 263:109263. doi:10.1016/j.vetmic.2021.109263 34749283

[45] Song Z, Yan T, Ran L, Niu Z, Zhang Y, Kan Z, Xu S, Zhang S, Zhang J, Zou H, Lei C. 2021. Reduced activity of intestinal surface Na^+^/H^+^ exchanger NHE3 is a key factor for induction of diarrhea after PEDV infection in neonatal piglets. Virology (Auckl) 563:64–73. doi:10.1016/j.virol.2021.08.011 34464882

[46] Yang Y, Yu Q, Song H, Ran L, Wang K, Xie L, Huang S, Niu Z, Zhang Y, Kan Z, Yan T, Song Z. 2020. Decreased NHE3 activity and trafficking in TGEV-infected IPEC-J2 cells via the SGLT1-mediated P38 MAPK/AKt2 pathway. Virus Res 280:197901. doi:10.1016/j.virusres.2020.197901 32070687 PMC7114662

[47] Yang Z, Ran L, Yuan P, Yang Y, Wang K, Xie L, Huang S, Liu J, Song Z. 2018. EGFR as a negative regulatory protein adjusts the activity and mobility of NHE3 in the cell membrane of IPEC-J2 cells with TGEV infection. Front Microbiol 9:2734. doi:10.3389/fmicb.2018.02734 30483239 PMC6243134

[48] Niu Z, Xu S, Zhang Y, Kan Z, Zhang J, Liu X, Zhang S, Zou H, Song Z. 2022. Transmissible gastroenteritis virus nucleocapsid protein interacts with Na+/H+ exchanger 3 to reduce Na+/H+ exchanger activity and promote piglet diarrhea. J Virol 96:e0147322. doi:10.1128/jvi.01473-22 36342433 PMC9682987

[49] Iwasaki M, Ngo N, de la Torre JC. 2014. Sodium hydrogen exchangers contribute to arenavirus cell entry. J Virol 88:643–654. doi:10.1128/JVI.02110-13 24173224 PMC3911747

[50] Wang D, Sun H, Lang F, Yun CC. 2005. Activation of NHE3 by dexamethasone requires phosphorylation of NHE3 at Ser663 by SGK1. Am J Physiol Cell Physiol 289:C802–C810. doi:10.1152/ajpcell.00597.2004 15888551 PMC1472807

[51] Wang D, Zhang H, Lang F, Yun CC. 2007. Acute activation of NHE3 by dexamethasone correlates with activation of SGK1 and requires a functional glucocorticoid receptor. Am J Physiol Cell Physiol 292:C396–C404. doi:10.1152/ajpcell.00345.2006 16971495 PMC2695591

[52] Lammers T, Sofias AM, van der Meel R, Schiffelers R, Storm G, Tacke F, Koschmieder S, Brümmendorf TH, Kiessling F, Metselaar JM. 2020. Dexamethasone nanomedicines for COVID-19. Nat Nanotechnol 15:622–624. doi:10.1038/s41565-020-0752-z 32747742 PMC7116110

[53] Horby P, Lim WS, Mafham M, Bell JL, Linsell L, Staplin N, Brightling C, Ustianowski A. 2021. Dexamethasone in hospitalized patients with Covid-19. N Engl J Med 2021 384:693–704. doi:10.1056/NEJMoa2021436 PMC7383595 32678530

[54] Tomazini BM, Maia IS, Cavalcanti AB, Berwanger O, Rosa RG, Veiga VC, Avezum A, Lopes RD, Bueno FR, Silva M. 2020. Effect of dexamethasone on days alive and ventilator-free in patients with moderate or severe acute respiratory distress syndrome and COVID-19: The CoDEX randomized clinical trial. JAMA 324:1307–1316. doi:10.1001/jama.2020.17021 32876695 PMC7489411

[55] Kino T, Burd I, Segars JH. 2021. Dexamethasone for severe COVID-19: How does it work at cellular and molecular levels? IJMS 22:6764. doi:10.3390/ijms22136764 34201797 PMC8269070

[56] Crothers K, DeFaccio R, Tate J, Alba PR, Goetz MB, Jones B, King JT Jr, Marconi V, Ohl ME, Rentsch CT, Rodriguez-Barradas MC, Shahrir S, Justice AC, Akgün KM. 2022. Dexamethasone in hospitalised COVID-19 patients not on intensive respiratory support. Eur Respir J 60:2102532. doi:10.1183/13993003.02532-2021 34824060 PMC8841623

[57] Sinha S, Rosin NL, Arora R, Labit E, Jaffer A, Cao L, Farias R, Nguyen AP, de Almeida LGN, Dufour A, Bromley A, McDonald B, Gillrie MR, Fritzler MJ, Yipp BG, Biernaskie J. 2022. Dexamethasone modulates immature neutrophils and interferon programming in severe COVID-19. Nat Med 28:201–211. doi:10.1038/s41591-021-01576-3 34782790 PMC8799469

[58] Zhang Y, Hu S, Wang J, Xue Z, Wang C, Wang N. 2021. Dexamethasone inhibits SARS-CoV-2 spike pseudotyped virus viropexis by binding to ACE2. Virology (Auckl) 554:83–88. doi:10.1016/j.virol.2020.12.001 PMC7744032 33387788

[59] Sarker H, Panigrahi R, Hardy E, Glover JNM, Elahi S, Fernandez-Patron C. 2022. Glucocorticoids bind to SARS-CoV-2 S1 at multiple sites causing cooperative inhibition of SARS-CoV-2 S1 interaction with ACE2. Front Immunol 13:906687. doi:10.3389/fimmu.2022.906687 35784352 PMC9242398

[60] Liu DX, Xu HY, Brown TD. 1997. Proteolytic processing of the coronavirus infectious bronchitis virus 1a polyprotein: identification of a 10-kilodalton polypeptide and determination of its cleavage sites. J Virol 71:1814–1820. doi:10.1128/JVI.71.3.1814-1820.1997 9032311 PMC191251

[61] Liao Y, Wang X, Huang M, Tam JP, Liu DX. 2011. Regulation of the p38 mitogen-activated protein kinase and dual-specificity phosphatase 1 feedback loop modulates the induction of interleukin 6 and 8 in cells infected with coronavirus infectious bronchitis virus. Virology (Auckl) 420:106–116. doi:10.1016/j.virol.2011.09.003 PMC7111953 21959016

[62] Wang H, Yuan X, Sun Y, Mao X, Meng C, Tan L, Song C, Qiu X, Ding C, Liao Y. 2019. Infectious bronchitis virus entry mainly depends on clathrin mediated endocytosis and requires classical endosomal/lysosomal system. Virology (Auckl) 528:118–136. doi:10.1016/j.virol.2018.12.012 PMC7111473 30597347

[63] Marwaha R, Sharma M. 2017. DQ-red BSA trafficking assay in cultured cells to assess cargo delivery to lysosomes. Bio Protoc 7:19. doi:10.21769/BioProtoc.2571 PMC5657473 29082291

[64] Feng SZ, Cao WS, Liao M. 2011. The PI3K/Akt pathway is involved in early infection of some exogenous avian leukosis viruses. J Gen Virol 92:1688–1697. doi:10.1099/vir.0.030866-0 21450945

[65] Choi JY, Kim SY, Son EJ, Kim JL, Shin JH, Song MH, Moon UY, Yoon JH. 2006. Dexamethasone increases fluid absorption via Na+/H+ exchanger (NHE) 3 activation in normal human middle ear epithelial cells. Eur J Pharmacol 536:12–18. doi:10.1016/j.ejphar.2006.02.031 16564041

[66] He P, Lee SJ, Lin S, Seidler U, Lang F, Fejes-Toth G, Naray-Fejes-Toth A, Yun CC. 2011. Serum- and glucocorticoid-induced kinase 3 in recycling endosomes mediates acute activation of Na+/H+ exchanger NHE3 by glucocorticoids. Mol Biol Cell 22:3812–3825. doi:10.1091/mbc.E11-04-0328 21865597 PMC3192861

[67] Kim MS, Lee SJ, Choi SH, Kang YJ, Kim KH. 2017. Dexamethasone treatment decreases replication of viral hemorrhagic septicemia virus in Epithelioma papulosum cyprini cells. Arch Virol 162:1387–1392. doi:10.1007/s00705-017-3248-x 28155193

[68] Lancz G, Whitaker-Dowling P, Marsh YV, Bradley G, Eppstein DA, Hackney JF, Hulick-Swardstrom B. 1990. Inhibition of vesicular stomatitis virus replication in dexamethasone-treated L929 cells. Proc Soc Exp Biol Med 193:190–196. doi:10.3181/00379727-193-43024 2154755

[69] Deng CX. 2020. Glucocorticoids save lives in COVID-19 patients. Int J Biol Sci 16:2477–2478. doi:10.7150/ijbs.49125 32760915 PMC7378644

[70] Ledford H. 2020. Coronavirus breakthrough: dexamethasone is first drug shown to save lives. Nature New Biol 582:469–469. doi:10.1038/d41586-020-01824-5 32546811

[71] Zhagn S, Li D, Chen H, Zheng D, Zhou Y, Chen B, Shi W, Lin R. 2020. Dynamic inflammatory response in a critically ill COVID-19 patient treated with corticosteroids. Zhejiang Da Xue Xue Bao Yi Xue Ban 49:220–226. doi:10.3785/j.issn.1008-9292.2020.03.10 32391668 PMC8800790

[72] Horby P, Lim WS, Bell M, Linsell JL, Staplin L, Brightling N, Ustianowski C, Elmahi E A. 2020. Dexamethasone in hospitalized patients with Covid-19. Preliminary Report. doi:10.1101/2020.06.22.20137273

[73] Bani-Sadr F, Hentzien M, Pascard M, N’Guyen Y, Servettaz A, Andreoletti L, Kanagaratnam L, Jolly D. 2020. Corticosteroid therapy for patients with COVID-19 pneumonia: a before-after study. Int J Antimicrob Agents 56:106077. doi:10.1016/j.ijantimicag.2020.106077 32634602 PMC7342082

[74] Solinas C, Perra L, Aiello M, Migliori E, Petrosillo N. 2020. A critical evaluation of glucocorticoids in the management of severe COVID-19. Cytokine Growth Factor Rev 54:8–23. doi:10.1016/j.cytogfr.2020.06.012 32616381 PMC7313507

[75] Villar J, Ferrando C, Martínez D, Ambrós A, Muñoz T, Soler JA, Aguilar G, Alba F, González-Higueras E, Conesa LA, Martín-Rodríguez C, Díaz-Domínguez FJ, Serna-Grande P, Rivas R, Ferreres J, Belda J, Capilla L, Tallet A, Añón JM, Fernández RL, González-Martín JM. 2020. Dexamethasone treatment for the acute respiratory distress syndrome: a multicentre, randomised controlled trial. Lancet Respir Med 8:267–276. doi:10.1016/S2213-2600(19)30417-5 32043986

[76] Taboada M, Caruezo V, Naveira A, Atanassoff PG. 2020. Corticosteroids and the hyper-inflammatory phase of the COVID-19 disease. J Clin Anesth 66:109926. doi:10.1016/j.jclinane.2020.109926 32474331 PMC7247969

[77] Cain DW, Cidlowski JA. 2020. After 62 years of regulating immunity, dexamethasone meets COVID-19. Nat Rev Immunol 20:587–588. doi:10.1038/s41577-020-00421-x 32778829 PMC7416654

[78] Li XW, Jiang RM, Guo JZ. 2003. Glucocorticoid in the treatment of severe acute respiratory syndrome patients: a preliminary report. Zhonghua Nei Ke Za Zhi. Vol. 42. 12895319

[79] Ali A, Ibrahim M, Eladl AH, Saif YM, Lee CW. 2013. Enhanced replication of swine influenza viruses in dexamethasone-treated juvenile and layer turkeys. Vet Microbiol 162:353–359. doi:10.1016/j.vetmic.2012.10.007 23123174

[80] Erlandsson AC, Bladh LG, Stierna P, Yucel-Lindberg T, Hammarsten O, Modéer T, Harmenberg J, Wikström AC. 2002. Herpes simplex virus type 1 infection and glucocorticoid treatment regulate viral yield, glucocorticoid receptor and NF-kappaB levels. J Endocrinol 175:165–176. doi:10.1677/joe.0.1750165 12379500

[81] Pechan P, Ardinger J, Ketavarapu J, Rubin H, Wadsworth SC, Scaria A. 2014. Aurintricarboxylic acid increases yield of HSV-1 vectors. Mol Ther Methods Clin Dev 1:6. doi:10.1038/mtm.2013.6 26015945 PMC4365865

[82] Hara Y, Shiraishi A, Kobayashi T, Kadota Y, Shirakata Y, Hashimoto K, Ohashi Y. 2009. Alteration of TLR3 pathways by glucocorticoids may be responsible for immunosusceptibility of human corneal epithelial cells to viral infections. Mol Vis 15:937–948. 19452017 PMC2683030

[83] Du T, Zhou G, Roizman B. 2012. Induction of apoptosis accelerates reactivation of latent HSV-1 in ganglionic organ cultures and replication in cell cultures. Proc Natl Acad Sci USA 109:14616–14621. doi:10.1073/pnas.1212661109 22908263 PMC3437834

[84] Parks WP, Scolnick EM, Kozikowski EH. 1974. Dexamethasone stimulation of murine mammary tumor virus expression: a tissue culture source of virus. Science 184:158–160. doi:10.1126/science.184.4133.158 4361099

[85] Indik S, Günzburg WH, Kulich P, Salmons B, Rouault F. 2007. Rapid spread of mouse mammary tumor virus in cultured human breast cells. Retrovirology (Auckl) 4:73. doi:10.1186/1742-4690-4-73 PMC2169256 17931409

[86] Solodushko V, Bitko V, Fouty B. 2009. Dexamethasone and mifepristone increase retroviral infectivity through different mechanisms. American Journal of Physiology-Lung Cellular and Molecular Physiology 297:L538–L545. doi:10.1152/ajplung.00162.2009 19561138 PMC2739773

[87] Singleton H, Graham SP, Frossard J-P, Bodman-Smith KB, Steinbach F. 2018. Infection of monocytes with European porcine reproductive and respiratory syndrome virus (PRRSV-1) strain Lena is significantly enhanced by dexamethasone and IL-10. Virology (Auckl) 517:199–207. doi:10.1016/j.virol.2018.02.017 29502802

[88] Lee GE, Shin CG. 2018. Influence of pretreatment with immunosuppressive drugs on viral proliferation. J Microbiol Biotechnol 28:1716–1722. doi:10.4014/jmb.1807.06054 30270601

[89] Petta I, Dejager L, Ballegeer M, Lievens S, Tavernier J, De Bosscher K, Libert C. 2016. The interactome of the glucocorticoid receptor and its influence on the actions of glucocorticoids in combatting inflammatory and infectious diseases. Microbiol Mol Biol Rev 80:495–522. doi:10.1128/MMBR.00064-15 27169854 PMC4867367

[90] Li Z, Zhao K, Lan Y, Lv X, Hu S, Guan J, Lu H, Zhang J, Shi J, Yang Y, Song D, Gao F, He W. 2017. Porcine hemagglutinating encephalomyelitis virus enters Neuro-2a cells via clathrin-mediated endocytosis in a Rab5-, cholesterol-, and pH-dependent manner. J Virol 91:23. doi:10.1128/JVI.01083-17 PMC5686734 28956766

[91] Takano T, Wakayama Y, Doki T. 2019. Endocytic pathway of feline coronavirus for cell entry: differences in serotype-dependent viral entry pathway. Pathogens 8:300. doi:10.3390/pathogens8040300 31888266 PMC6963708

[92] Cavanagh D. 2007. Coronavirus avian infectious bronchitis virus. Vet Res 38:281–297. doi:10.1051/vetres:2006055 17296157

[93] Yuan X, Zhang X, Wang H, Mao X, Sun Y, Tan L, Song C, Qiu X, Ding C, Liao Y. 2023. The ubiquitin-proteasome system facilitates membrane fusion and uncoating during coronavirus entry. Viruses 15:2001. doi:10.3390/v15102001 37896778 PMC10610886

[94] Eifart P, Ludwig K, Böttcher C, Haan CA, Rottier PJ, Korte T, Herrmann A. 2007. Role of endocytosis and low pH in murine hepatitis virus strain A59 cell entry. J Virol 81:10758–10768. doi:10.1128/JVI.00725-07 17626088 PMC2045462

[95] Li D, Cavanagh D. 1992. Coronavirus IBV-induced membrane fusion occurs at near-neutral pH. Arch Virol 122:307–316. doi:10.1007/BF01317192 1309994 PMC7086749

[96] Gao J, Gui M, Xiang Y. 2020. Structural intermediates in the low pH-induced transition of influenza hemagglutinin. PLoS Pathog 16:e1009062. doi:10.1371/journal.ppat.1009062 33253316 PMC7728236

[97] Wiley DC, Skehel JJ. 1987. The structure and function of the hemagglutinin membrane glycoprotein of influenza virus. Annu Rev Biochem 56:365–394. doi:10.1146/annurev.bi.56.070187.002053 3304138

[98] Sieczkarski SB, Whittaker GR. 2003. Differential requirements of Rab5 and Rab7 for endocytosis of influenza and other enveloped viruses. Traffic 4:333–343. doi:10.1034/j.1600-0854.2003.00090.x 12713661

[99] Niu Z, Xu S, Zhang Y, Kan Z, Zhang J, Liu X, Zhang S, Zou H, Song Z. 2022. Transmissible gastroenteritis virus nucleocapsid protein interacts with Na(+)/H(+) exchanger 3 to reduce Na(+)/H(+) exchanger activity and promote piglet diarrhea. J Virol 96:e0147322. doi:10.1128/jvi.01473-22 36342433 PMC9682987

[100] Dai J, Wang H, Liao Y, Tan L, Sun Y, Song C, Liu W, Ding C, Luo T, Qiu X. 2022. Non-targeted metabolomic analysis of chicken kidneys in response to coronavirus IBV infection under stress induced by dexamethasone. Front Cell Infect Microbiol 12:945865. doi:10.3389/fcimb.2022.945865 35909955 PMC9335950

[101] Kan-O K, Ramirez R, MacDonald MI, Rolph M, Rudd PA, Spann KM, Mahalingam S, Bardin PG, Thomas BJ. 2017. Human metapneumovirus infection in chronic obstructive pulmonary disease: Impact of glucocorticosteroids and interferon. J Infect Dis 215:1536–1545. doi:10.1093/infdis/jix167 28379462

[102] Chan JW, Zhang AJ, Chan CS, Yip CY, Mak WN, Zhu H, Poon VM, Tee KM, Zhu Z, Cai JP, Tsang JL, Chik KH, Yin F, Chan KH, Kok KH, Jin DY, Au-Yeung RH, Yuen KY. 2016. Zika virus infection in dexamethasone-immunosuppressed mice demonstrating disseminated infection with multi-organ involvement including orchitis effectively treated by recombinant type I interferons. EBioMedicine 14:112–122. doi:10.1016/j.ebiom.2016.11.017 27884655 PMC5161441

[103] Mazer MB, Davitt E, Turnbull IR, Caldwell CC, Brakenridge SC, Remy KE, Hotchkiss RS. 2021. In vitro-administered dexamethasone suppresses T cell function with reversal by interleukin-7 in coronavirus disease 2019. Crit Care Explor 3:e0378. doi:10.1097/CCE.0000000000000378 33834168 PMC8021361

[104] Wang H, Liu D, Sun Y, Meng C, Tan L, Song C, Qiu X, Liu W, Ding C, Ying L. 2021. Upregulation of DUSP6 impairs infectious bronchitis virus replication by negatively regulating ERK pathway and promoting apoptosis. Vet Res 52:7. doi:10.1186/s13567-020-00866-x 33431056 PMC7798014

[105] Gong Y, Tang N, Liu P, Sun Y, Lu S, Liu W, Tan L, Song C, Qiu X, Liao Y, Yu S, Liu X, Lin SH, Ding C. 2022. Newcastle disease virus degrades SIRT3 via PINK1-PRKN-dependent mitophagy to reprogram energy metabolism in infected cells. Autophagy 18:1503–1521. doi:10.1080/15548627.2021.1990515 34720029 PMC9298456

[106] Liu P, Yin Y, Gong Y, Qiu X, Sun Y, Tan L, Song C, Liu W, Liao Y, Meng C, Ding C. 2019. In vitro and in vivo metabolomic profiling after infection with virulent Newcastle disease virus. Viruses 11:962. doi:10.3390/v11100962 31635316 PMC6832399

[107] Gao B, Gong X, Fang S, Weng W, Wang H, Chu H, Sun Y, Meng C, Tan L, Song C, Qiu X, Liu W, Forlenza M, Ding C, Liao Y. 2021. Inhibition of anti-viral stress granule formation by coronavirus endoribonuclease nsp15 ensures efficient virus replication. PLoS Pathog 17:e1008690. doi:10.1371/journal.ppat.1008690 33635931 PMC7946191

[108] Gao B, Gong X, Fang S, Weng W, Wang H, Chu H, Sun Y, Meng C, Tan L, Song C, Qiu X, Liu W, Forlenza M, Ding C, Liao Y. 2021 b. Inhibition of anti-viral stress granule formation by coronavirus endoribonuclease nsp15 ensures efficient virus replication. PLoS Pathog 17:e1008690. doi:10.1371/journal.ppat.1008690 33635931 PMC7946191

[109] Wu W, Qu Y, Yu S, Wang S, Yin Y, Liu Q, Meng C, Liao Y, Ur Rehman Z, Tan L, Song C, Qiu X, Liu W, Ding C, Sun Y. 2021. Caspase-dependent cleavage of DDX21 suppresses host innate immunity. MBio 12:e0100521. doi:10.1128/mBio.01005-21 34125604 PMC8262918

[110] Kan X, Yin Y, Song C, Tan L, Qiu X, Liao Y, Liu W, Meng S, Sun Y, Ding C. 2021. Newcastle-disease-virus-induced ferroptosis through nutrient deprivation and ferritinophagy in tumor cells. iScience 24:102837. doi:10.1016/j.isci.2021.102837 34368653 PMC8326413

[111] Liu W, Qiu X, Song C, Sun Y, Meng C, Liao Y, Tan L, Ding Z, Liu X, Ding C. 2018. Deep sequencing-based transcriptome profiling reveals avian interferon-stimulated genes and provides comprehensive insight into Newcastle disease virus-induced host responses. Viruses 10:162. doi:10.3390/v10040162 29601508 PMC5923456

[112] Dai J, Wang H, Liao Y, Tan L, Sun Y, Song C, Liu W, Qiu X, Ding C. 2022. RNA-seq and LC-MS/MS analysis of antiviral effects mediated by cold stress and stress hormone corticosterone in chicken DF-1 cells. Vet Microbiol 275:109580. doi:10.1016/j.vetmic.2022.109580 36308941

[113] Qiu X, Yu Y, Yu S, Zhan Y, Wei N, Song C, Sun Y, Tan L, Ding C. 2014. Development of strand-specific real-time RT-PCR to distinguish viral RNAs during Newcastle disease virus infection. ScientificWorldJournal 2014:934851. doi:10.1155/2014/934851 25379553 PMC4212552

[114] El-Hage N, Rodriguez M, Dever SM, Masvekar RR, Gewirtz DA, Shacka JJ. 2015. HIV-1 and morphine regulation of autophagy in microglia: limited interactions in the context of HIV-1 infection and opioid abuse. J Virol 89:1024–1035. doi:10.1128/JVI.02022-14 25355898 PMC4300622

[115] Undem C, Rios EJ, Maylor J, Shimoda LA. 2012. Endothelin-1 augments Na⁺/H⁺ exchange activity in murine pulmonary arterial smooth muscle cells via Rho kinase. PLoS ONE 7:e46303. doi:10.1371/journal.pone.0046303 23029469 PMC3460862

[116] Scott DA, Docampo R, Benchimol M. 1998. Analysis of the uptake of the fluorescent marker 2’,7’-bis-(2-carboxyethyl)-5(and-6)-carboxyfluorescein (BCECF) by hydrogenosomes in Trichomonas vaginalis. Eur J Cell Biol 76:139–145. doi:10.1016/S0171-9335(98)80027-7 9696354

