# Supplementary material for: Dexamethasone disrupts intracellular pH homeostasis to delay coronavirus infectious bronchitis virus cell entry via sodium hydrogen exchanger 3 activation
Source: J Virol. 2025 May 9;99(6):e01894-24. doi: 10.1128/jvi.01894-24 (PMC12172481; doi:10.1128/jvi.01894-24)
Supplement: Figure S1 — qRT-PCR was performed to evaluate the effects of Dex on IBV. [file jvi.01894-24-s0001.docx]

**Supplemental figure 1.**


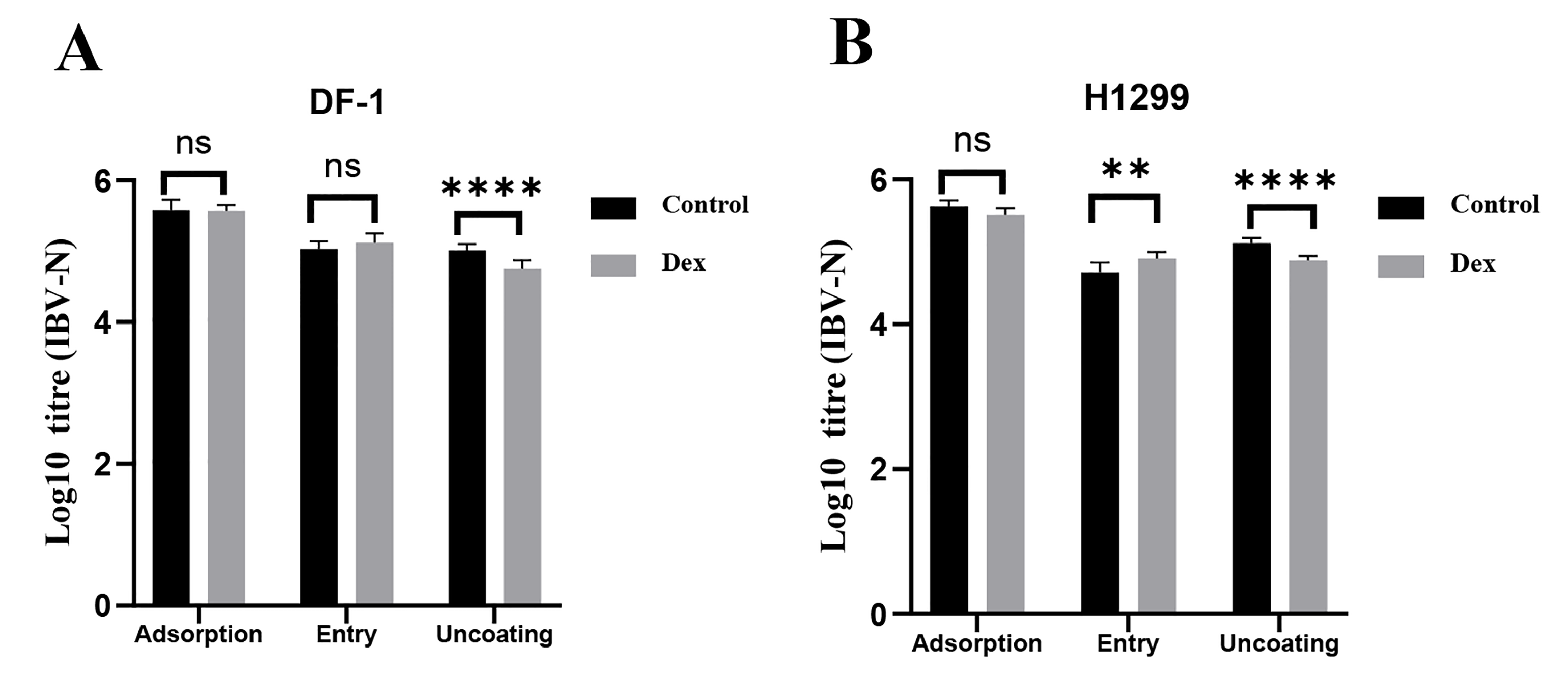


**Figure S1. qRT-PCR was performed to evaluate the effects of Dex on IBV** **adsorption, entry, and uncoating.** DF-1 (A) and H1299 (B) cells were pretreated with Dex (10μg/mL) for 24 h and then infected with IBV (MOI=10). Incubate at 4℃ for 1h (as 0hpi), then transfer to 37℃ for 1h (as1hpi) and 3h (as 3hpi). Cells were harvested at 0 hpi (adsorption), 1 hpi(entry), and 3 hpi (uncoating) to determined IBV-N mRNA levels using absolute qRT-PCR.
