# Supplementary material for: Dexamethasone disrupts intracellular pH homeostasis to delay coronavirus infectious bronchitis virus cell entry via sodium hydrogen exchanger 3 activation
Source: J Virol. 2025 May 9;99(6):e01894-24. doi: 10.1128/jvi.01894-24 (PMC12172481; doi:10.1128/jvi.01894-24)
Supplement: Figure S2 — Protonex Red 600 pH fluorescent probe was performed to evaluate the effects of Dex on pH in endo-lysosomes. [file jvi.01894-24-s0002.docx]

**Supplemental figure 2.**

**
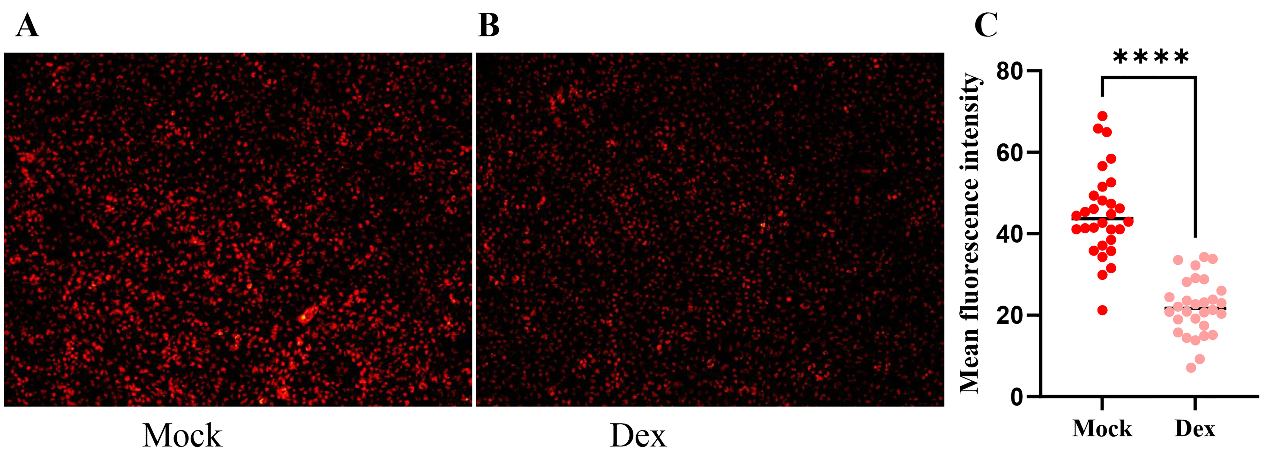
**

**Figure S2.** **Protonex Red 600 pH fluorescent probe was performed to evaluate the effects of Dex on pH in endo-lysosomes.** DF-1 cells were pretreated with Dex (10μg/mL) for 24 h and then Protonex Red 600 pH fluorescent probe was performed to evaluate the pH in endo-lysosomes (A), Dex-untreated cells were used as controls (B). Representative single-plane fluorescence microscope of DF-1 cells showing the Protonex Red 600 fluorescence (red). Average fluorescence intensity was measured using ImageJ software (N=30) (C).
