# Supplementary material for: Dexamethasone disrupts intracellular pH homeostasis to delay coronavirus infectious bronchitis virus cell entry via sodium hydrogen exchanger 3 activation
Source: J Virol. 2025 May 9;99(6):e01894-24. doi: 10.1128/jvi.01894-24 (PMC12172481; doi:10.1128/jvi.01894-24)
Supplement: Figure S3 — CCK-8 was used to evaluate the cell activity. [file jvi.01894-24-s0003.docx]

**Supplemental figure 3.**


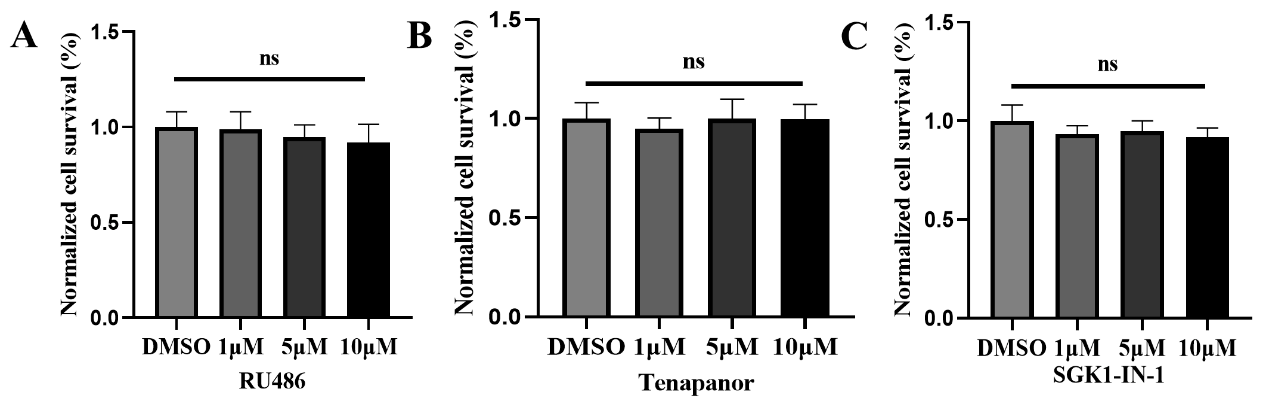


**Figure S3.** **Cell counting kit-8 (CCK-8) was used to evaluate the cell activity.** Cell counting kit-8 (CCK-8) was used to evaluate the effects of RU486(A), Tenapanor(B), and SGK1-IN-1(C) on cell activity.
