# Supplementary material for: Dexamethasone disrupts intracellular pH homeostasis to delay coronavirus infectious bronchitis virus cell entry via sodium hydrogen exchanger 3 activation
Source: J Virol. 2025 May 9;99(6):e01894-24. doi: 10.1128/jvi.01894-24 (PMC12172481; doi:10.1128/jvi.01894-24)
Supplement: Figure S4 — Dex effects on Na+/H+ exchange in H1299 cell membranes. [file jvi.01894-24-s0004.docx]

**Supplemental figure 4.**


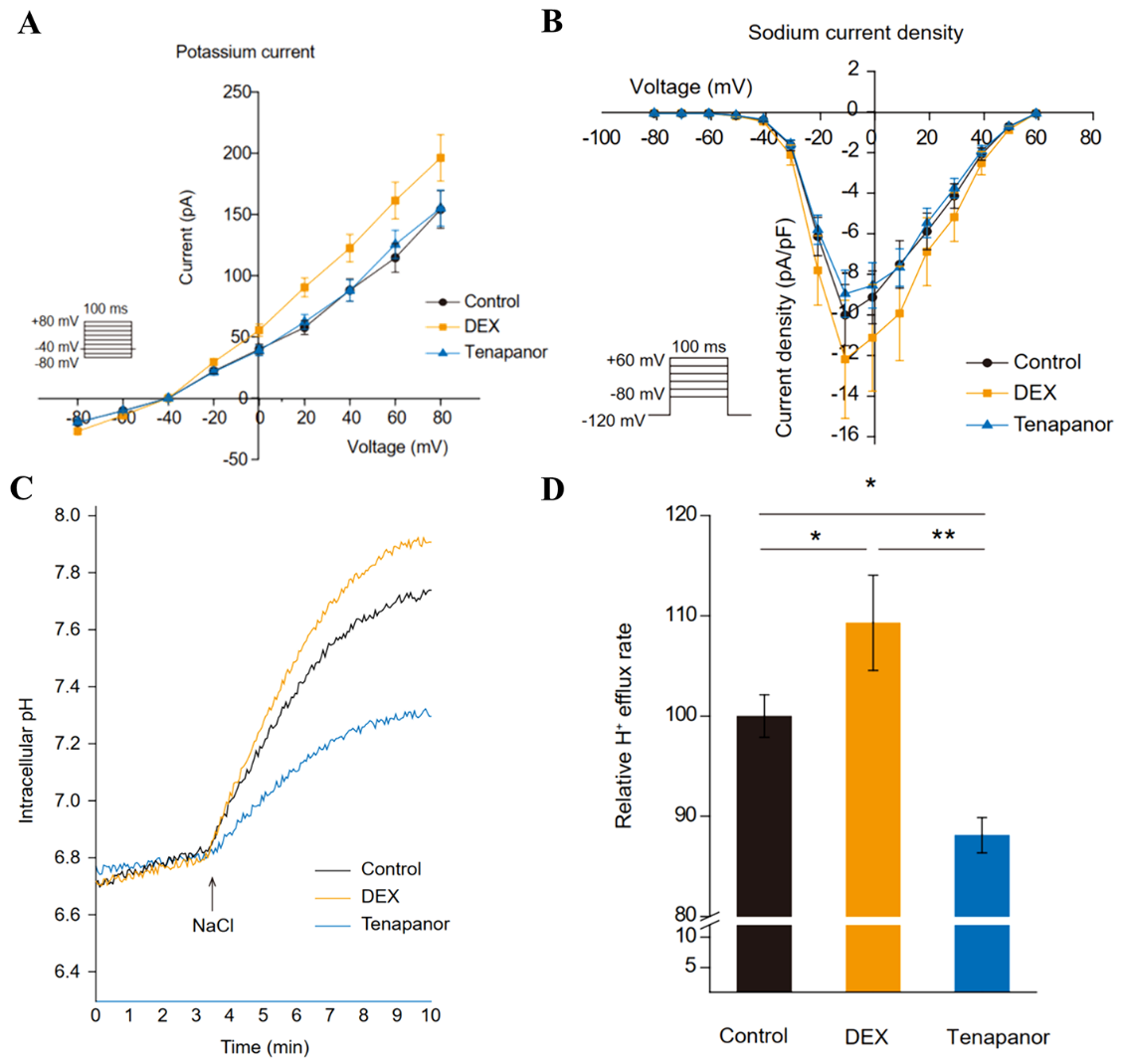


**Figure S4.** **Dex effects on Na+/H+ exchange in H1299 cell membranes.** (A) Patch clamp test was used to detect the effects of Dex and Tenapanor on cell membranes K^+^ current density. (B) Patch clamp test was used to detect the effects of Dex and Tenapanor on cell membranes Na^+^ current density. (C) BCECF-AM probe detects intracellular pH changes to evaluate NHE3 activity. (D) Statistical analysis of cell membranes relative H^+^ efflux rate.
