# Supplementary material for: Dexamethasone disrupts intracellular pH homeostasis to delay coronavirus infectious bronchitis virus cell entry via sodium hydrogen exchanger 3 activation
Source: J Virol. 2025 May 9;99(6):e01894-24. doi: 10.1128/jvi.01894-24 (PMC12172481; doi:10.1128/jvi.01894-24)
Supplement: Figure S5 — Dex and Tenapanor effects on intracellular pH levels in Vero cells. [file jvi.01894-24-s0005.docx]

**Supplemental figure 5.**


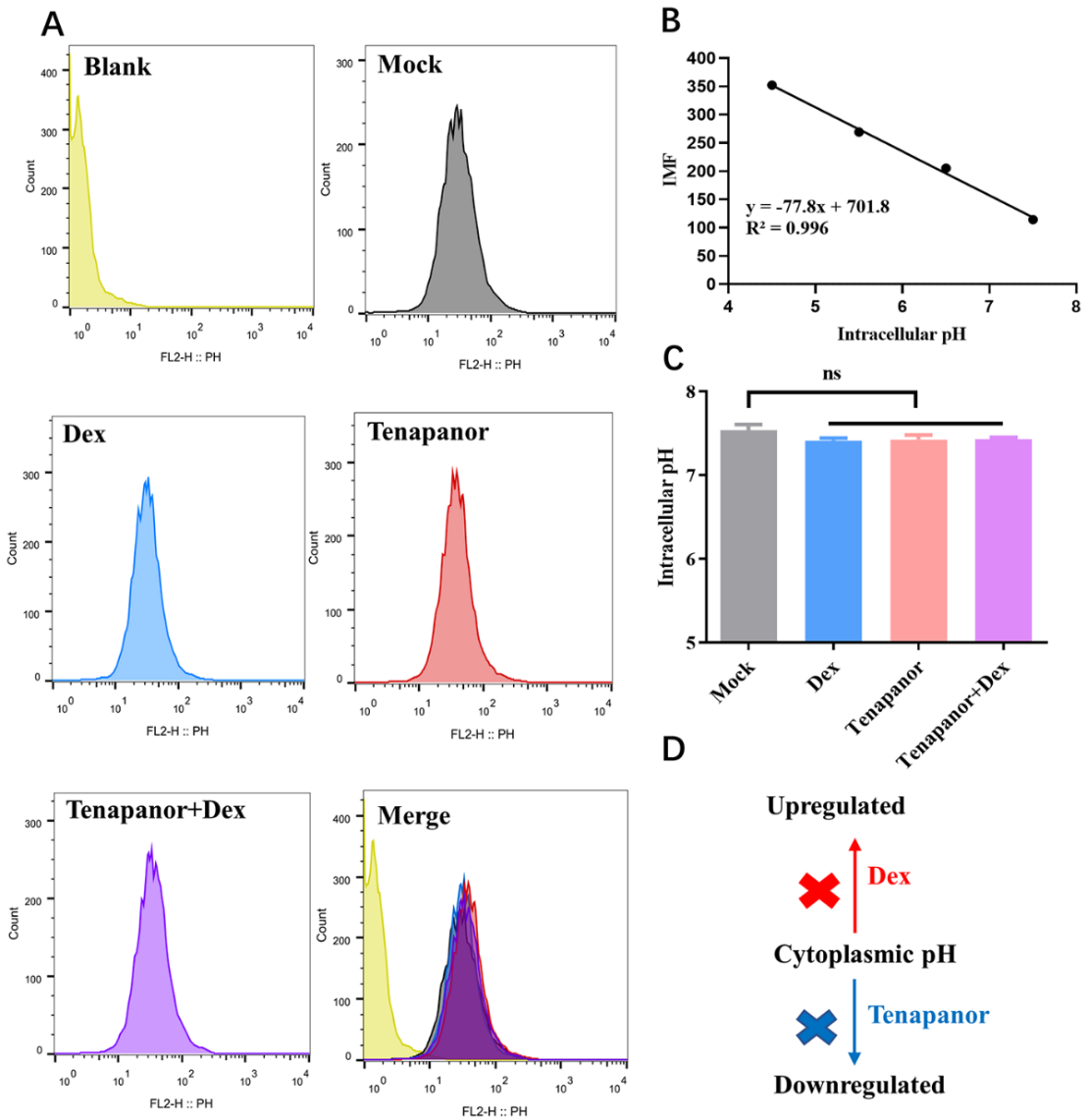


**Figure S5. Dex and Tenapanor effects on intracellular pH levels in Vero cells.** Vero cells were treated with DMEM medium containing Dex (100 μg/mL) or Tenapanor (10 μM). The experiment was divided into four groups: Mock group (blank DMEM medium), Dex group (Dex treatment for 12h), Tenapanor group (Tenapanor treatment for 2h), Tenapanor+Dex group (Tenapanor treatment for 2h first, followed by Dex treatment for 12h). (A) Intracellular pH in Vero cells was determined by flow cytometry and pHrodo™ Red. (B) A standard curve was established during flow cytometry to determine a linear relationship between intracellular pH and the average pHrodo™ Red fluorescence intensity. (C) Histograms show intracellular pH quantification in Vero cells following Dex and Tenapanor treatment (flow cytometry). (D) The effects of Dex and Tenaparor on intracellular pH regulation.
