# Supplementary material for: Dexamethasone disrupts intracellular pH homeostasis to delay coronavirus infectious bronchitis virus cell entry via sodium hydrogen exchanger 3 activation
Source: J Virol. 2025 May 9;99(6):e01894-24. doi: 10.1128/jvi.01894-24 (PMC12172481; doi:10.1128/jvi.01894-24)
Supplement: Figure S6 — Construction and validation of SLC9A3 gene knockout cell line. [file jvi.01894-24-s0006.docx]

**Supplemental figure 6.**


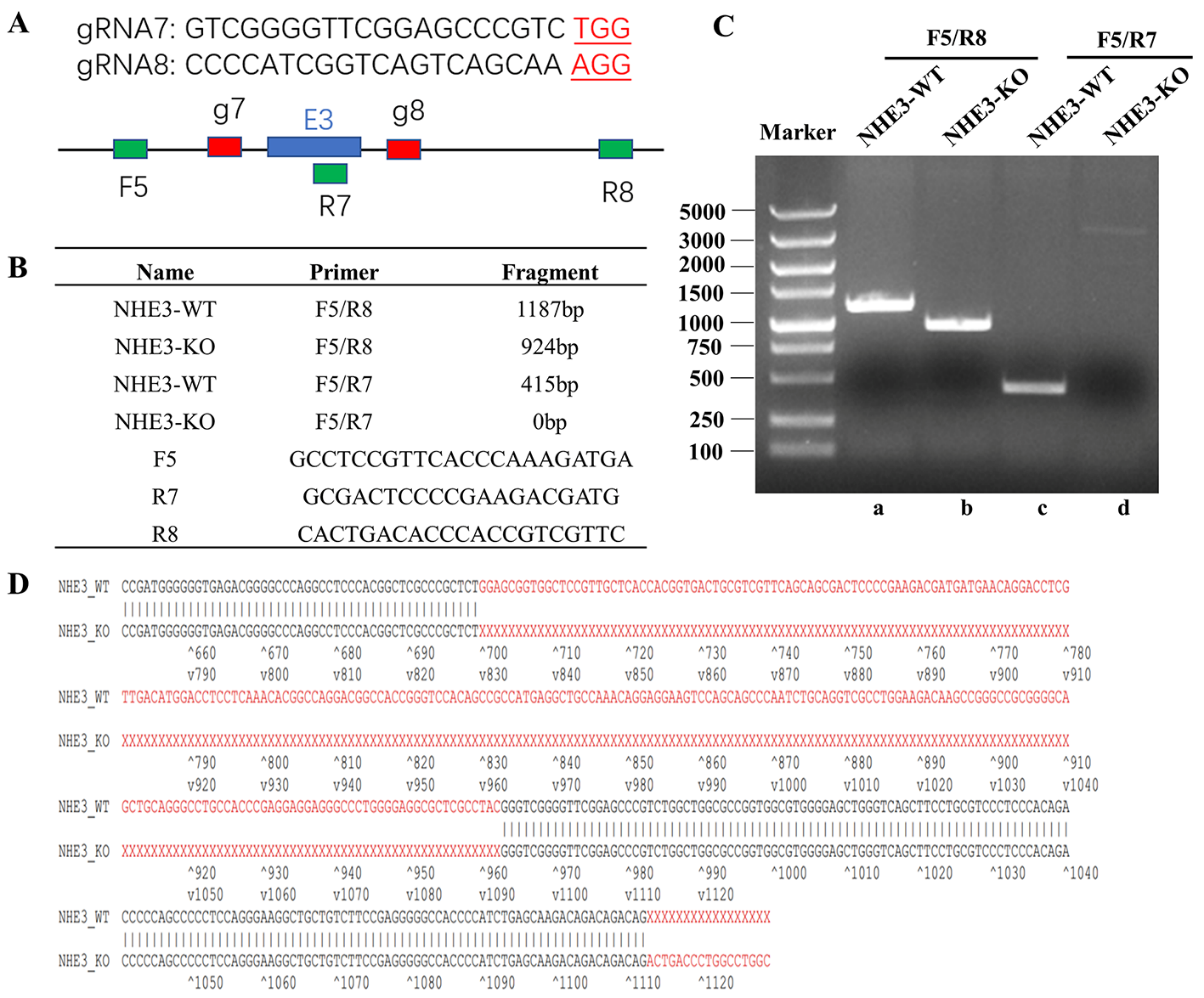


**Figure S6. Construction and validation of *SLC9A3* gene knockout cell line using CRISPR/Cas9 technique.** (A) The sgRNAs sequence designed by the online CRISPR design tool. (B) Knock out the location of the region and identify the primer sequence. (C). Positive clones were identified by PCR. D. PCR products were sequenced and identified.
