# Supplementary material for: Dexamethasone disrupts intracellular pH homeostasis to delay coronavirus infectious bronchitis virus cell entry via sodium hydrogen exchanger 3 activation
Source: J Virol. 2025 May 9;99(6):e01894-24. doi: 10.1128/jvi.01894-24 (PMC12172481; doi:10.1128/jvi.01894-24)
Supplement: Table S1 — All qRT-PCR primers used in this study. [file jvi.01894-24-s0007.docx]

**Table S1. All qRT-PCR primers used in this study.**

| **Temlate** | **Primers** | **Sequences (5’-3’)** |
| --- | --- | --- |
| IBV RNA (gRNA(-)) | qIBV-F | TTAGTAGAACCAACAAACACGACAG |
|  | qIBV-R | TTTAGCAGAACATTTTGACGCAGAT |
| IBV(N gene) | qIBV-F | CAGAAGAAGGGCTCTCGCATTAC |
|  | qIBV-R | AGGTTGAGCATTGCCGTAACAC |
| H9N2(NP gene) | qH9N2-F | ACCAGTGCATGGAGACAATTC |
|  | qH9N2-R | CAAATGTTGCATCTGCAAGAC |
| H9N2(M gene) | qH9N2-F | TCTCACAGACAGATGGCGACTACC |
|  | qH9N2-R | GCTTCTGCTGCCTGCTCACTC |
| NDV(NP gene) | qNDV-F | AGTGATGTGCTCGGACCTTC |
|  | qNDV-R | CCTGAGGAGAGGCATTTGCTA |
| β-Actin（chicken） | Actin-F | GAGAAATTGTGCGTGACATCA |
|  | Actin-R | CCTGAACCTCTCATTGCCA |
